# Fast Delayed Emission in New Pyridazine-Based Compounds

**DOI:** 10.3389/fchem.2020.572862

**Published:** 2021-01-07

**Authors:** Simonas Krotkus, Tomas Matulaitis, Stefan Diesing, Graeme Copley, Emily Archer, Changmin Keum, David B. Cordes, Alexandra M. Z. Slawin, Malte C. Gather, Eli Zysman-Colman, Ifor D. W. Samuel

**Affiliations:** ^1^Organic Semiconductor Centre, Scottish Universities Physics Alliance (SUPA), School of Physics and Astronomy, University of St. Andrews, St Andrews, United Kingdom; ^2^Organic Semiconductor Centre, EaStCHEM School of Chemistry, University of St. Andrews, St Andrews, United Kingdom

**Keywords:** thermally activated delayed fluorescence (TADF), pyridazine, organic light-emitting diode (OLED), reverse intersystem crossing (rISC), donor-acceptor (D–A) architecture

## Abstract

Three novel donor-acceptor molecules comprising the underexplored pyridazine (Pydz) acceptor moiety have been synthesized and their structural, electrochemical and photophysical properties thoroughly characterized. Combining Pydz with two phenoxazine donor units linked via a phenyl bridge in a *meta* configuration (**dPXZMePydz**) leads to high reverse intersystem crossing rate *k*_RISC_ = 3.9 · 10^6^ s^−1^ and fast thermally activated delayed fluorescence (TADF) with <500 ns delayed emission lifetime. Efficient triplet harvesting via the TADF mechanism is demonstrated in OLEDs using **dPXZMePydz** as the emitter but does not occur for compounds bearing weaker donor units.

**Graphical Abstract:**
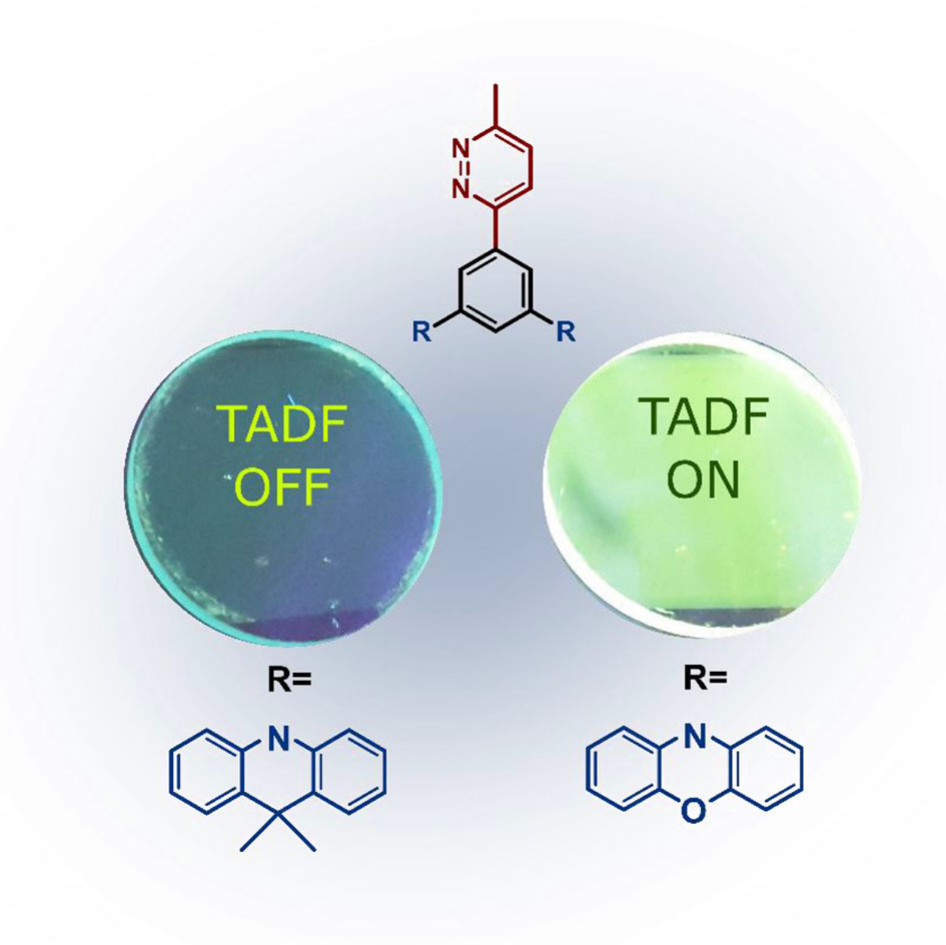
Thin film luminecence under UV light of pyridazine-based emitters, dPXZMePydz **(left)** and dCzMePydz **(right)**.

## Introduction

Electroluminescence (EL) from organic light-emitting diodes (OLEDs) originates from the radiative decay of neutral excited states (excitons) formed by the recombination of holes and electrons. Spin statistics dictate that three quarters of the excitons created in working OLEDs are triplets. Since the ground state of most organic luminophores is singlet, considerable effort has been concentrated on development of materials that exhibit fast spin conversion from triplet to singlet states. The first breakthrough in EL efficiency was thus achieved by introducing organometallic phosphorescent emitters (Baldo et al., 1998; Adachi et al., 2001). The heavy atom effect in these materials enhances spin-orbit coupling, promoting phosphorescence with microsecond lifetime, and, importantly external quantum efficiencies (EQEs) that surpass the limit of 5% for fluorescent materials. However, the most commonly used organometallic complexes are based on iridium(III) and platinum(II) and these materials are not sustainable given their ultra-low abundance in the Earth's crust. While efficient and stable red and green phosphorescent OLEDs are now commercialized, and encouraging pure blue phosphorescent OLEDs were demonstrated recently (Lee et al., 2016; Li et al., 2018; Pal et al., 2018), blue device stability remains an unresolved issue (Yang et al., 2015; Jacquemin and Escudero, 2017). For these reasons, alternative routes to harvest both singlet and triplet states using purely organic emitters via delayed fluorescence channels, namely, triplet-triplet annihilation (TTA) (Kondakov et al., 2009) and thermally activated delayed fluorescence (TADF) (Uoyama et al., 2012; Wong and Zysman-Colman, 2017), are being hotly pursued. TADF is based on triplet up-conversion to the first excited singlet level via reverse intersystem crossing (rISC). For the rate of this process (*k*_rISC_) to be sufficiently high to outcompete the non-radiative internal conversion of the triplet state, a small singlet-triplet splitting energy ΔE_ST_ is required, which is achieved in intramolecular charge transfer (ICT) states present in molecules composed of electron-donating (D) and electron-accepting (A) units. Concomitantly, high photoluminescence quantum yield (Φ_PL_) is needed in high performance emitters for OLEDs. Indeed, competitively efficient TADF OLEDs based on D-A emitter architectures have now been reported for all relevant display colors (Tao et al., 2014). An ongoing research effort is also aimed at addressing issues related to device stability and the link to the structure of the emitters. In particular, the design of emitters that show fast *k*_rISC_ and short delayed fluorescence lifetimes is desired in order to mitigate against bimolecular interactions, such as TTA and triplet-polaron annihilation. This is currently pursued via controlled molecular fine-tuning of the spatial separation and twist angles between D and A units, as well as D/A unit strength (Milián-Medina and Gierschner, 2012; Tanaka et al., 2013; Im et al., 2017).

**Figure 1:**
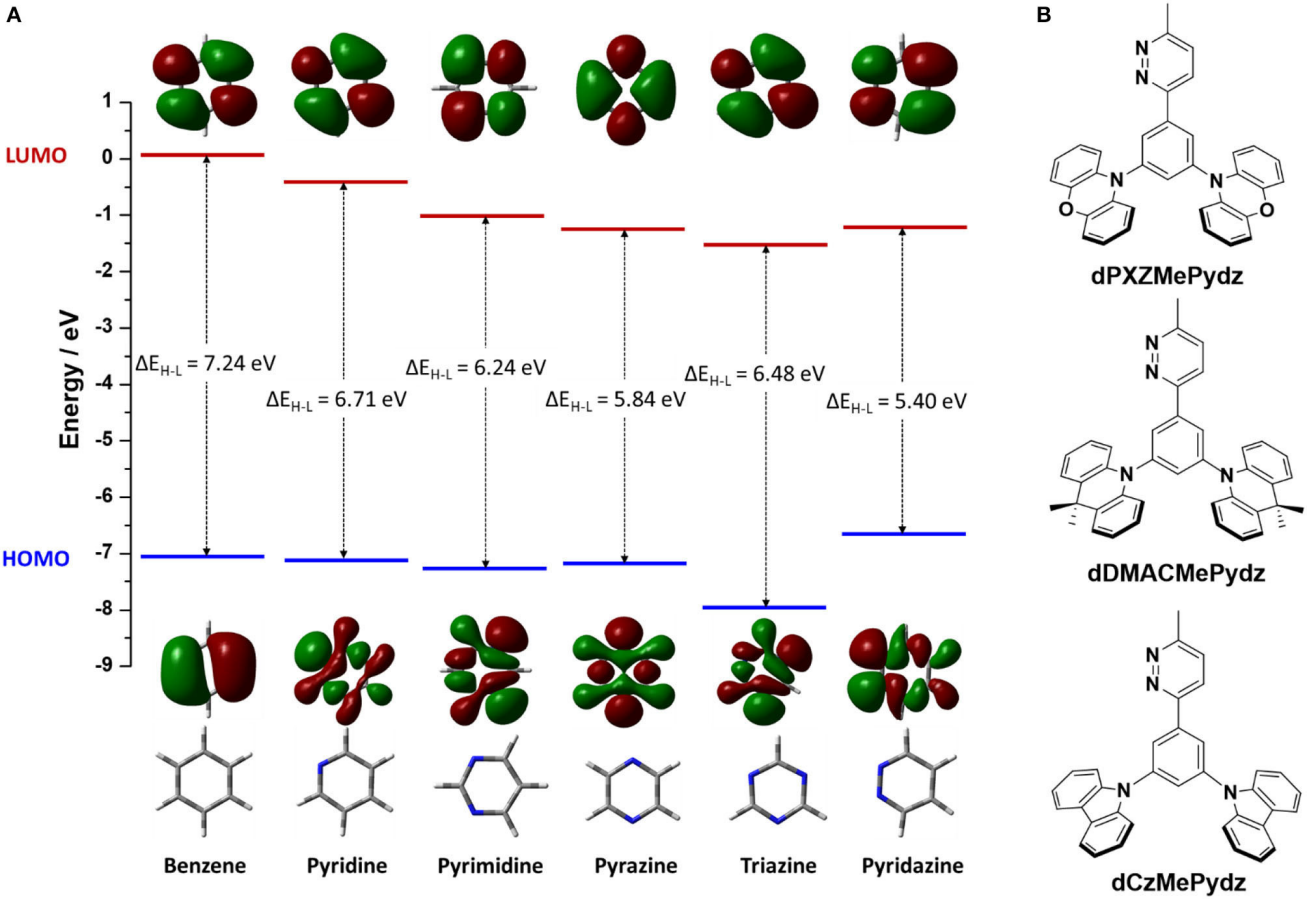
**(A)** Structure of benzene and other azines, together with their corresponding HOMO and LUMO electron density distributions (isovalue = 0.02) and energy levels, calculated at PBE0/6-31G(d,p) level in vacuum. **(B)** Chemical structures of compounds with pyridazine acceptor.

Among the *N*-heterocyclic electron-acceptors, 1,3,5-triazine is one of the most employed (Wong and Zysman-Colman, 2017; Huang et al., 2019; Sharma et al., 2019; Wang et al., 2019; Woo et al., 2019). Other heterocycles used in this role include pyridines (Rajamalli et al., 2017, 2018a,b, 2019), pyrimidines (Komatsu et al., 2016; Nakao et al., 2017; dos Santos et al., 2019), and pyrazines (Figure 1A) (dos Santos et al., 2019; Kato et al., 2019; Liu et al., 2019). Missing from this family of heterocycles is pyridazine (Pydz), which is investigated in this work as an acceptor in three novel D-A compounds (Figure 1B). Pyridazine-based compounds have been employed as ligands in phosphorescent emitters (Guo et al., 2015; Zhang et al., 2016), as well as hosts for phosphorescent OLEDs (Jia et al., 2019), and as fluorescent probes (Qu et al., 2020). To date, there are no examples of pyridazine-based TADF emitters used in OLEDs.

## Results and Discussion

### Synthesis

To evaluate the electron-accepting ability of pyridazine it is essential to consider the energy level of its lowest unoccupied molecular orbital (LUMO). Density functional theory (DFT) calculated LUMO levels of benzene and related *N*-heterocycles predict that pyridazine possesses a comparable LUMO level to those of triazine and pyrazine (Figure 1A) (Ortiz et al., 2009; Liu et al., 2017; Jin et al., 2019). Pyridazine possesses a more destabilized highest occupied molecular orbital (HOMO) as compared to other studied azines, thus yielding the smallest HOMO-LUMO gap, ΔE_H−L_. Substituted pyridazines are most easily synthesized at the three and six positions (i.e., *ortho* to the nitrogen atoms). We thus envisaged the three targeted emitters in Figure 1B containing a phenylene bridge, which was expected to induce a certain degree of N···H intramolecular hydrogen bonding between the Pydz and the phenylene and a co-planar conformation. Decoration of the phenylene with electron donors of different strength connected at the *meta* positions would strongly electronically decouple these groups from the Pydz acceptor, leading to potential TADF emitters.

Each of the three emitters in this study comprised a 3-methyl-pyridazine (MePydz) acceptor moiety linked by a phenylene bridge to two identical donor units disposed in a *meta* configuration with respect to the position of the acceptor (Figure 1B). The donor units chosen were phenoxazine (PXZ, compound **dPXZMePydz**), 9,9-dimethyl-9,10-dihydroacridine (DMAC, **dDMACMePydz**), and carbazole (Cz, **dCzMePydz**). By weakening the donor strength from PXZ to DMAC to Cz we expected to systematically shift the emission energy of the compounds toward the blue. Conjugation between donor and acceptor groups should be greatly reduced owing to their *meta* disposition about the central phenylene. As such, the exchange integral between the HOMO and the LUMO will be small, leading to a correspondingly small singlet-triplet energy gap, ΔE_ST_, between the lowest singlet and triplet excited states, which is desired for an efficient TADF mechanism.

**Scheme 1:**
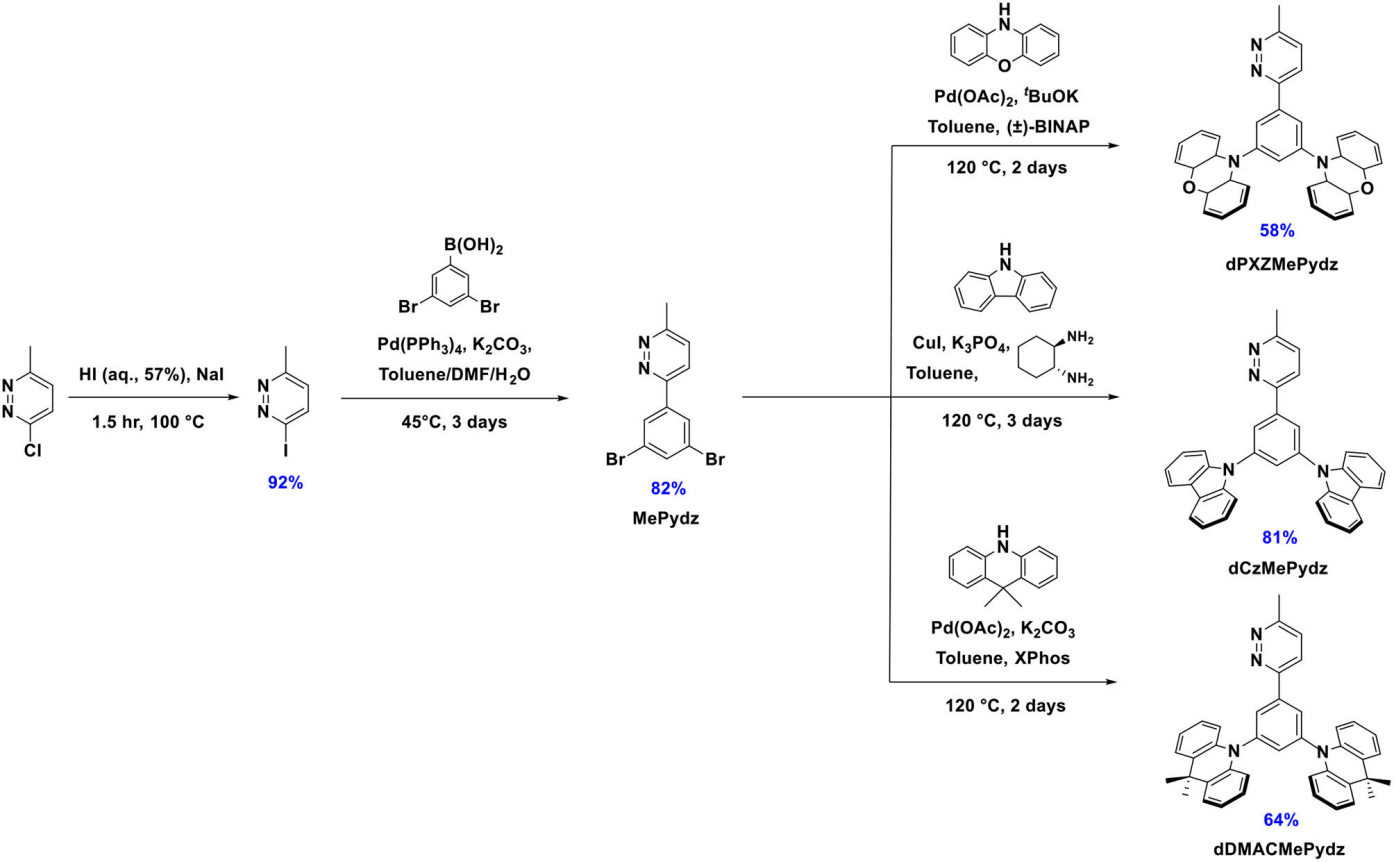
Synthetic scheme of pyridazine-based emitters.

The synthesis of the three pyridazine-containing compounds is shown in Scheme 1. Key intermediate MePydz was prepared by Suzuki-Miyaura (Suzuki, 2005) cross-coupling reaction between 3-iodo-6-methylpyridazine and (3,5-dibromophenyl)boronic acid. Coupling of MePydz to PXZ, DMAC and Cz under either Buchwald-Hartwig (Forero-Cortés and Haydl, 2019) or modified Ullmann coupling (Antilla et al., 2004) conditions yielded the target materials **dPXZMePydz**, **dDMACMePydz**, and **dCzMePydz**, respectively, in average to high yields. The chemical structures of the three derivatives were identified by a combination of ^1^H, ^13^C NMR spectroscopy (Supplementary Figures 1–8), high resolution mass spectrometry (Supplementary Figures 9–11) and elemental analysis. The compounds were purified by column chromatography, and purity was ascertained by HPLC (Supplementary Figures 12–14) and elemental analysis (Supplementary Figures 15–17).

### Structural Characterization

**Figure 2:**
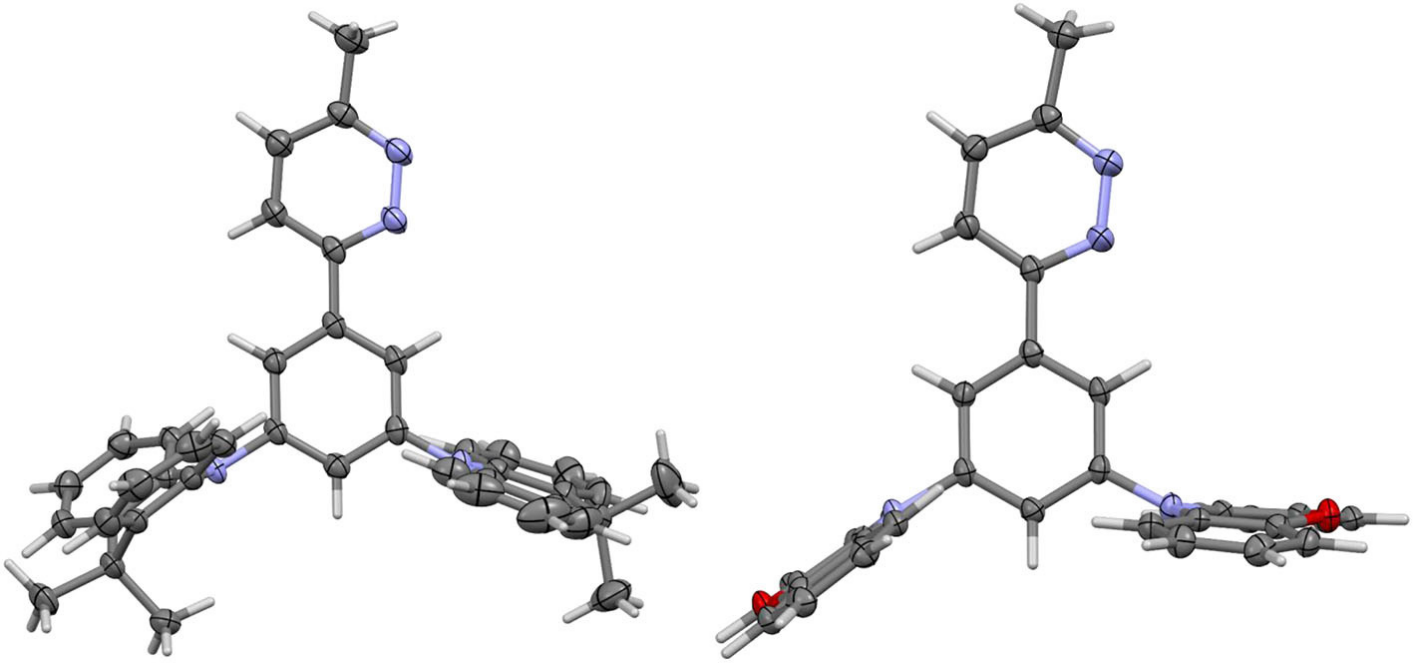
Thermal ellipsoid plots of the crystal structures of **(left) dDMACMePydz** and **(right)** one independent molecule of **dPXZMePydz**. Ellipsoids are drawn at the 50% probability level.

The structures of **dPXZMePydz** and **dDMACMePydz** were also confirmed by single crystal X-ray diffraction analysis (Figure 2, Supplementary Table 1). The structure of **dPXZMePydz** contained two independent molecules, one of which showed a similar pyrazine-phenylene geometry to **dDMACMePydz**, the other of which showed a different geometry. The dihedral angle between the pyridazine and the adjacent phenylene was found to be relatively small for **dDMACMePydz** and the equivalent **dPXZMePydz** (13.12 and 11.37°, respectively), although a much greater angle was seen for the second independent molecule in **dPXZMePydz** (42.63°), while in all cases, the mean planes of the donor groups are disposed close to orthogonal with respect to the plane of the phenylene bridge (66.41 – 80.86°). The DMAC donor *anti* to the diazine in **dDMACMePydz** was found to have a 38.51° pucker angle across its central ring, the other adopting a flat conformation (3.57° pucker). In contrast, none of the PXZ donors in **dPXZMePydz** showed the same extent of ring-pucker. In one independent molecule the diazine-facing PXZ adopted a flatter conformation than the *anti* donor group (6.12 vs. 14.11°), while in the second, the diazine-facing PXZ was more puckered than the *anti* donor (11.88 vs. 4.88°). The other significant geometric difference between the structures is in the degree of pyramidalisation of the nitrogen of the donor groups. In **dDMACMePydz** both donor nitrogens showed similar slight degrees of pyramidalisation (C_phenylene_-N···CMe_2_ 169.00 and 171.82°), but in **dPXZMePydz** each independent molecule showed different patterns of nitrogen pyramidalisation. One of these showed a more pyramidal nitrogen in the *anti* donor group (C_phenylene_-N···O 160.52 and 179.51, for *anti* and *syn* donors groups, respectively), whereas the other showed a more pyramidal nitrogen in the *syn* donor group (C_phenylene_···N···O 174.46 and 155.26, for *anti* and *syn* donors groups, respectively).

### Theoretical Calculations

**Figure 3:**
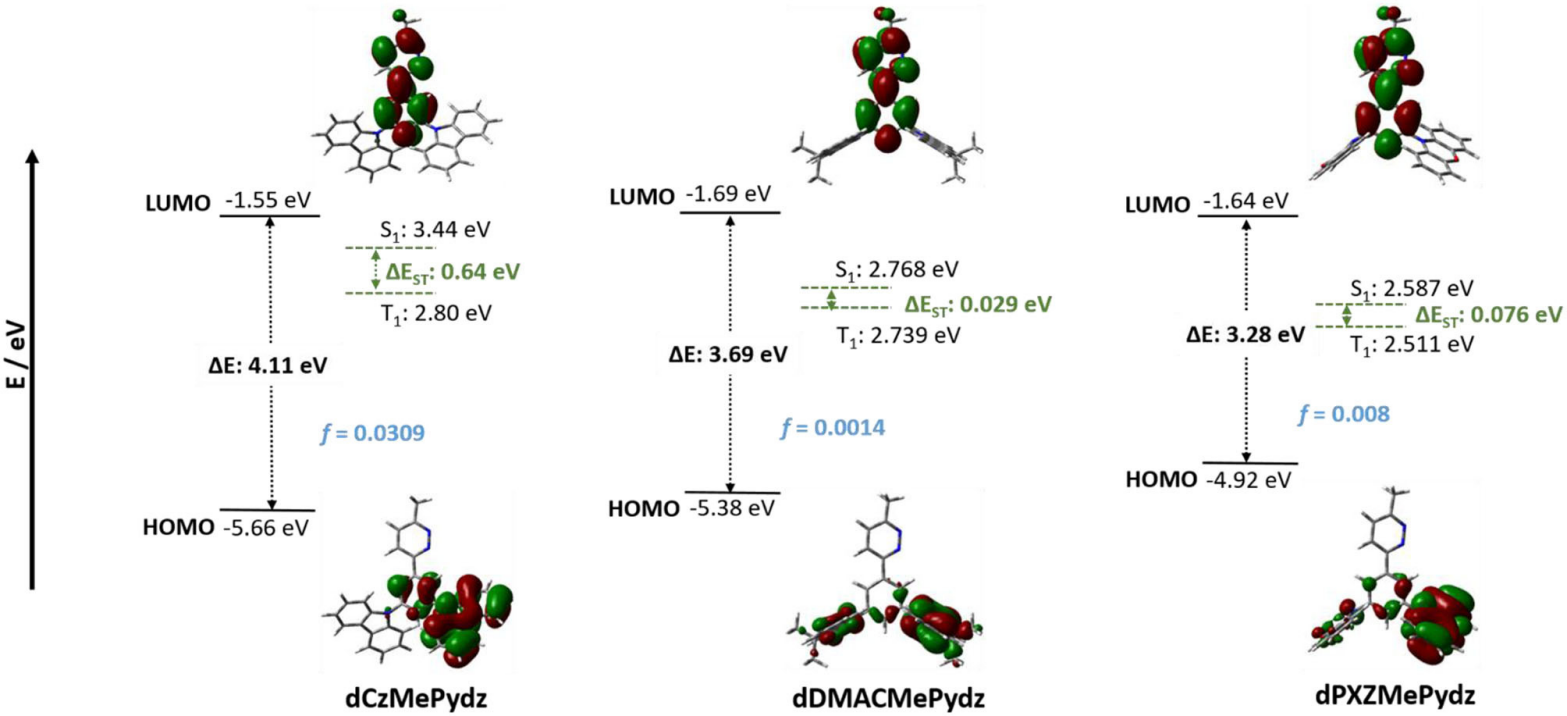
Frontier molecular orbitals (isovalue = 0.02) and corresponding energy values calculated at PBE0/6-31G(d,p) level in vacuum.

DFT calculated energies of the first excited singlet and triplet states, S_1_ and T_1_, respectively, and the HOMO and LUMO levels for the three compounds are shown in Figure 3 along with the electron-density distribution of the HOMO and LUMO orbitals. The HOMOs for **dPXZMePydz** and **dDMACMePydz** are localized on the donor moieties with only a minor contribution associated with the phenylene bridge, while for **dCzMePydz** the HOMO is delocalized over the phenylene bridge and one of the two carbazole groups. The multi-donor design should lead to a degeneracy of frontier orbitals. This is evidenced by the HOMO-1 being close in energy (~70–200 meV) to HOMO in each of the compounds (see Supplementary Figure 19). The HOMO levels reflect the strength of the donor moieties with values of −4.92, −5.38, and −5.66 eV for **dPXZMePydz**, **dDMACMePydz**, and **dCzMePydz**, respectively. The LUMOs for all three compounds are delocalized along the **MePydz** acceptor and phenylene bridge. The trend in LUMO energies mirrors that observed for the HOMO levels, but the influence of the donor on the LUMO level is significantly attenuated, with LUMO levels of −1.64, −1.69, and −1.55 eV for **dPXZMePydz**, **dDMACMePydz**, and **dCzMePydz**, respectively. The ΔE_H−L_ values thus increase from 3.28 to 3.69 to 4.11 eV for **dPXZMePydz**, **dDMACMePydz**, and **dCzMePydz**, respectively. The Tamm-Dancoff approximation to time-dependent DFT calculations predict the transition from the ground state to the lowest excited state (S_0_-S_1_) to have ICT character for all three molecules. The S_0_-T_1_ transitions of **dDMACMePydz** and **dPXZMePydz** are likewise CT in nature, while the S_0_-T_1_ transition of **dCzMePydz** possesses local exciton (LE) character within the acceptor moiety. The calculated energies and oscillator strengths for the transitions from ground state to S_1_,_2_ and T_1,2,3,4_ levels are summarized in Supplementary Table 2. For **dPXZMePydz**, the S_1_ state is at 2.58 eV, while it increases in energy to 2.77 eV and 3.44 eV for **dDMACMePydz** and **dCzMePydz**, respectively. Thus, as expected, a blue-shift in emission is predicted with the decreasing donor unit strength. The frontier molecular orbitals of **dPXZMePydz** and **dDMACMePydz** are well-separated. This is reflected in the small calculated oscillator strengths (*f* = 8.0·10^−3^ and 1.4·10^−3^, respectively) as well as small singlet-triplet splitting energies (ΔE_ST_ = 76 and 29 meV, respectively). On the other hand, there is significant HOMO-LUMO overlap in **dCzMePydz**, which is reflected in much larger *f* of 3.1·10^−2^ and large ΔE_ST_ of 635 meV. Thus, this latter compound would not be expected to exhibit efficient TADF while the other two emitters would. The T_2_ and T_3_ states of **dPXZMePydz** are energetically very close to the S_2_ state (ΔE_S2T2_ = 41 meV and ΔE_S2T3_ = 9 meV, respectively, see Supplementary Figure 20, Supplementary Table 2). For **dDMACMePydz** there is a similar alignment of higher lying singlet and triplet excited states (ΔE_S1T2_ = −35 meV, ΔE_S2T3_ = 33 meV), while for **dCzMePydz** only T_4_ is close to S_1_ (ΔE_S1T4_ = −15 meV). However, it is unlikely that **dCzMePydz** would undergo triplet exciton harvesting from T_4_ since internal conversion proceeding via T_3_ and T_2_ is highly likely to trap triplet excitons in the T_1_ state.

### Electrochemistry

**Figure 4:**
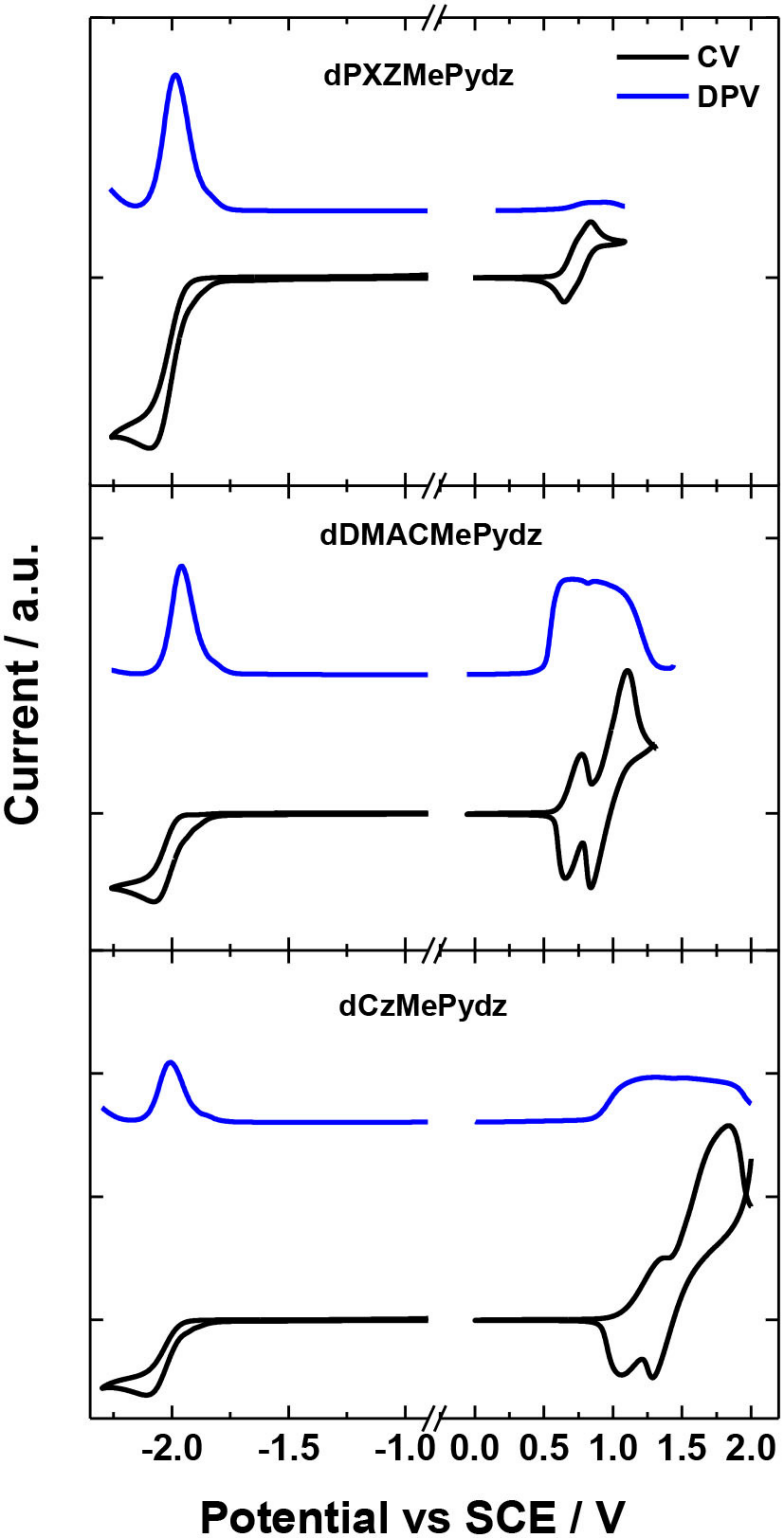
CV and DPV curves of **dPXZMePydz**, **dDMACMePydz**, and **dCzMePydz**. Oxidation measured in DCM and reduction in DMF (scan rate: 0.03 V/s).

Figure 4 and Table 1 summarize the electrochemical characterization of the compounds. Cyclic and differential pulse voltammetry (CV and DPV, respectively) were employed in order to elucidate the energy levels of the studied materials. The oxidation potentials were 0.74 V, 0.71 V, and 1.21 V for the **dPXZMePydz**, **dDMACMePydz**, and **dCzMePydz**, respectively. Only **dPXZMePydz** demonstrated reversible oxidation, while other two compounds show quasi-reversible oxidation. As expected, the use of the weaker Cz electron donor in **dCzMePydz** resulted in a significantly more anodic oxidation potential, which is consistent with oxidation of other carbazole-based emitters (Kukhta et al., 2017). The ${\text{E}}_{\text{PA}}^{\text{OX}}$ for **dDMACMePydz** is cathodically shifted by only 0.03 V compared to that of **dPXZMePydz**, thereby indicating that within these emitters DMAC and PXZ possess comparable donating strength. All three compounds demonstrated almost identical reduction potentials with E_RED_ values of −1.93 V, −1.94 V, and −1.94 V for the **dPXZMePydz**, **dDMACMePydz**, and **dCzMePydz**, respectively. Ionization potential and electron affinity values calculated based on oxidation and reduction potentials obtained from the DPV experiments follow the same trends. Expectedly, the HOMO level is destabilized with increasing strength of the donor with ionization potential values of 5.22 eV for **dCzMePydz**, 4.60 eV for **dDMACMePydz** and 4.78 eV for **dPXZMePydz**. The nature of the donor has no notable effect on the LUMO levels, with electron affinity values of 1.96 eV, 1.98 eV, and 1.93 eV for **dPXZMePydz**, **dDMACMePydz**, and **dCzMePydz**, respectively. These results indicate essentially no electronic coupling between the donor and acceptor moieties. As a result, the electrochemical bandgap is systematically increased as a function of weakening donor strength along the DMAC to PXZ to Cz.

**Table 1:** Summary of electrochemical properties.

**Compounds**	***E^***OX***^*_**PA**_/**	***E^**RED**^*_**PC**_/**	**IP/**	**EA/**	**Δ*E*_**REDOX**_/**
	**V vs. SCE^a^**	**V vs. SCE^a^**	**eV^b^**	**eV^b^**	**eV^c^**
**dPXZMePydz**	0.74 (0.85)	−1.93 (−1.98)	4.78	1.96	2.82
**dDMACMePydz**	0.71 (0.67)	−1.94 (−1.96)	4.60	1.98	2.62
**dCzMePydz**	1.21 (1.29)	−1.94 (−2.01)	5.22	1.93	3.29

a *Oxidation potentials (E^OX^_PA_) were estimated in DCM, while reduction potentials (E^RED^_PC_) were estimated in DMF. Values in parentheses are the data obtained by DPV and are vs. SCE (Fc/Fc^+^ = 0.46 V in DCM and 0.45 V in DMF). CV scan rate 100 mV/s*.

b *Ionization potential and electron affinity values were determined by DPV using formula IP/EA = -(E_OX/RED_ + 4.8) eV*.

c *ΔE_REDOX_ = |IP – EA|*.

### Photophysics

**Figure 5:**
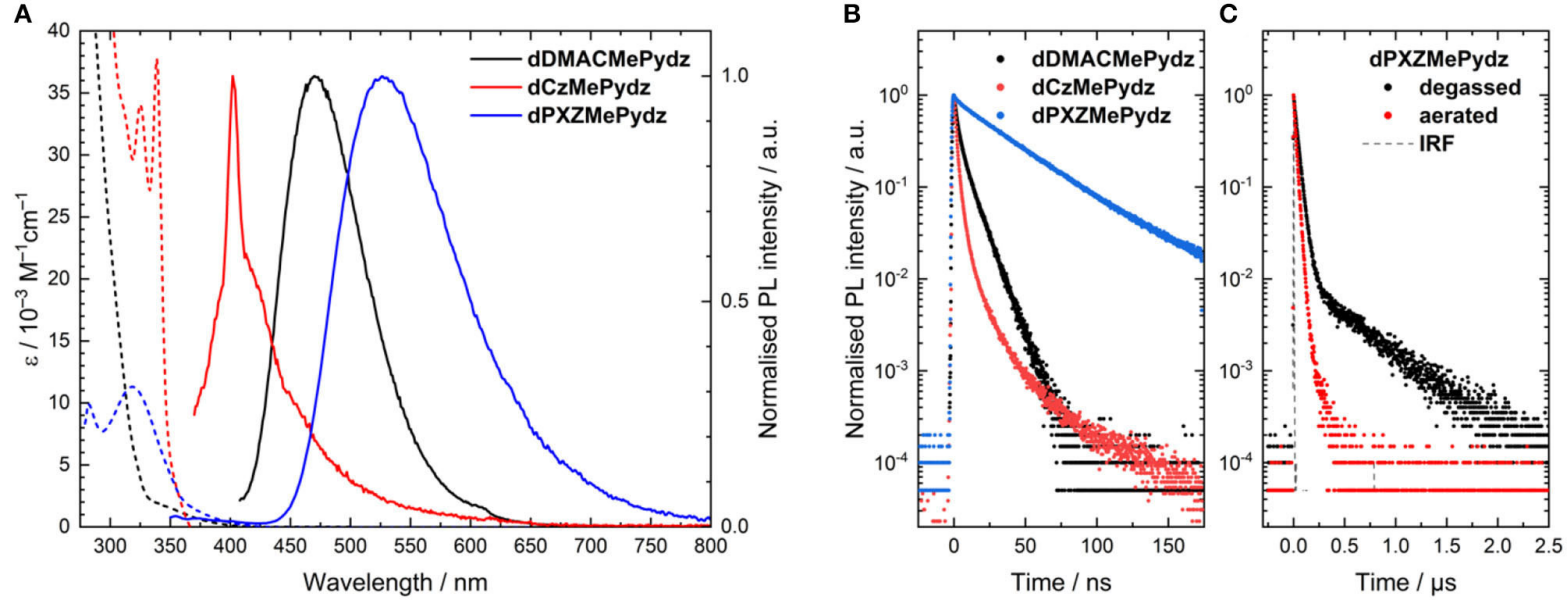
**(A)** Solution absorption (dashed lines) and emission spectra (solid lines) of the pyridazine-based compounds in toluene (λ_exc_ = 300 nm for **dDMACMePydz**, λ_exc_ = 360 nm for **dCzMePydz**, λ_exc_ = 315 nm for **dPXZMePydz**). Peak at 400 nm in emission spectra of **dCzMePydz** is an instrument artifact; **(B)** Time resolved photoluminescence spectra in degassed toluene (λ_exc_ = 378 nm); **(C)** Time resolved photoluminescence of **dPXZMePydz** in aerated and degassed toluene (λ_exc_ = 378 nm) at room temperature.

The absorption spectra in toluene are shown in Figure 5. All spectra resemble those of the donor units used, with the absorption spectrum of **dPXZMePydz** having a more notable lowest energy band located at 370–450 nm. A hypsochromic shift in the absorption onset is observed (λ_abs_ = 445 nm in **dPXZMePydz**, λ_abs_ = 415 nm for **dDMACMePydz**, and λ_abs_ = 360 nm for **dCzMePydz**). Weak and structureless lower energy absorption bands are attributed to the ICT transition between the D and A units, in line with the trends observed in theoretically simulated absorption spectra (Supplementary Figure 18). Predicted absorption spectra for **dDMACMePydz** and **dPXZMePydz** contain low energy bands located between 400 nm and 480 nm, which are composed of S_0−1,2_ excitations, which are of the same ICT character, just involving the other donor (Supplementary Table 2, Supplementary Figure 19). In the case of **dCzMePydz**, the S_0−1,2_ excitations are also of ICT character; however, they are in competition with similar energy local excitations (S_0−3_) in the acceptor moiety.

The steady-state photoluminescence (PL) spectra in toluene (Figure 5A) also show the same trend as that found in the absorption spectra, with a progressive red-shift in the emission maxima as a function of Cz>DMAC>PXZ (Table 2). The red-shift of emission is accompanied by an increase in Φ_PL_. **dCzMePydz** exhibits a Φ_PL_ value of 1.0%, compared to 4.5% for **dDMACMePydz** and 8.5% for **dPXZMePydz**. The time-resolved PL of **dPXZMePydz** and **dDMACMePydz** (Figure 5B) show a mono-exponential prompt component with lifetimes of τ_p_ = 39 ns and 7 ns, respectively. The prompt component of the PL of **dCzMePydz** has a multiexponential form with an average lifetime of τ_p_ = 2 ns. The transient PL of **dPXZMePydz** shows a remarkably fast delayed emission with a lifetime of τ_d_ = 0.47 μs in degassed solution. Such a short delayed emission lifetime of **dPXZMePydz** is a result of modular design strategy. Use of *meta* disposition of D and A moieties around the central phenylene bridge was previously shown to produce emitters showing high ICT character for their S_1_ and T_1_ states, small ΔE_ST_, as well as short τ_d_ and fast *k*_rISC_ (Wong et al., 2018; Kukhta et al., 2019). The delayed component is quenched upon exposing the solution to air, thus implicating accessible triplet states and corroborating that emission in this compound proceeds via TADF. For both **dDMACMePydz** and **dCzMePydz** no delayed PL component could be detected in toluene.

**Table 2:** Summary of photophysical properties in solution and thin film (40 nm).

**Compound**	**Toluene**	**15 wt% doped DPEPO films**						
	**λ_PL_/nm^a^**	**Φ_PL_/%^b^**	**τ_p_/ns^c^**	**τ_d_/μs^c^**	**λ_PL_/nm^a^**	**Φ_PL_/%^b^**	**τ_p_/ns^c^**	**τ_d_/μs^c^**
**dPXZMePydz**	525	8.5	39	0.47	520	13	40.9 (88.5 %)	1.43 (11.5 %)
**dDMACMePydz**	475	4.5	7		470	2.2 (1.3)	10.7 (86.9 %), 61.2 (12.6%)	1.69 (0.5 %)
**dCzMePydz**	405	1.0	2^d^		-	e	-	-

a *λ_exc_ = 330 nm*.

b *Measured under a N_2_ atmosphere. Values in parentheses represent measurements in air*.

c *λ_exc_ = 378 nm*.

d *Average lifetime*.

e *The Φ_PL_ film measurement intensity was too low to quote a value*.

**Figure 6:**
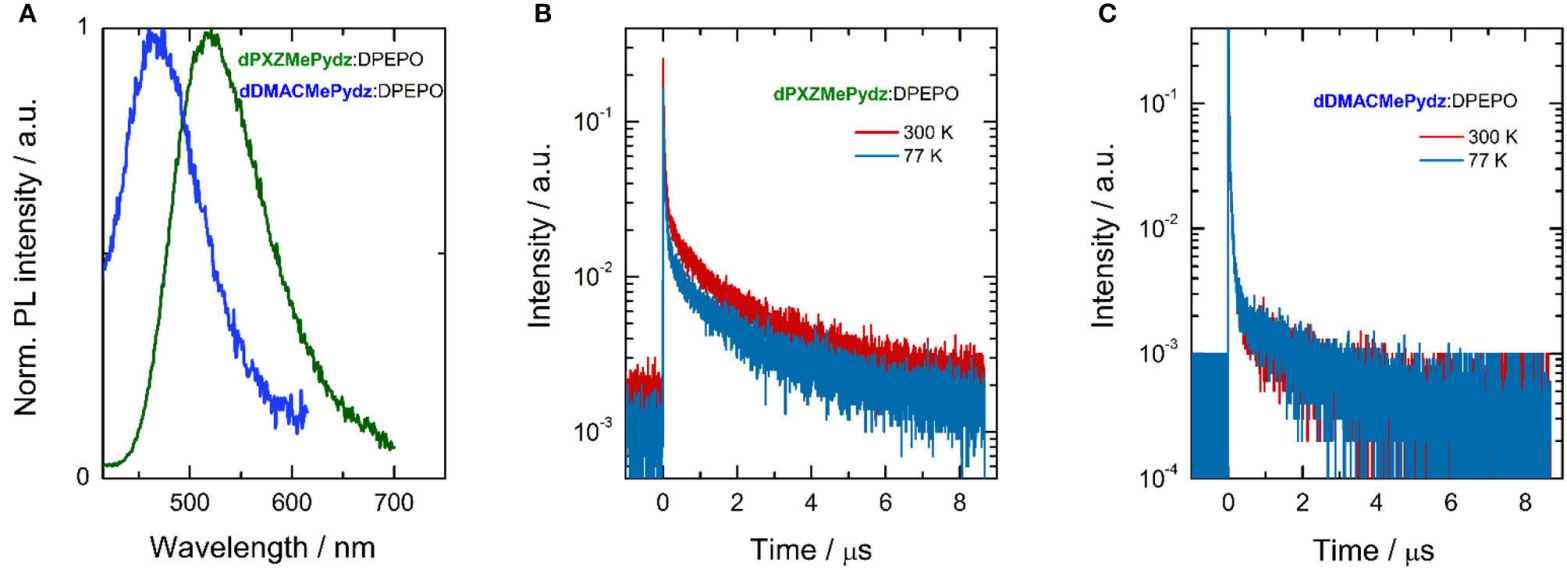
Steady-state and time-resolved photoluminescence of thin films; **(A)** integrated emission spectra (λ_exc_ = 330 nm); time-resolved PL (λ_exc_ = 378 nm) of **(B) dPXZMePydz**:DPEPO (15 wt%), and **(C) dDMACMePydz**:DPEPO (15 wt%) at 300 K (red solid line) and 77 K (blue solid line).

We next assessed the photophysical properties of the emitters in a polar, wide band-gap host, bis[2-(diphenylphosphino)phenyl]ether oxide (DPEPO). Doped thin films of the emitters in DPEPO were produced via vacuum deposition. **dPXZMePydz** and **dDMACMePydz** were investigated only, due to their higher Φ_PL_ values in solution and their greater likelihood of being TADF materials based on the DFT calculations. The optimal emitter concentration was found to be around 15 wt%. Slightly blue-shifted emission (λ_PL_ = 520 and 470 nm for **dPXZMePydz** and **dDMACMePydz**, respectively) was recorded compared to those measured in toluene (Figure 6A). **dPXZMePydz** exhibited a slightly higher Φ_PL_ of 11% in DPEPO film while **dDMACMePydz** showed a slightly lower Φ_PL_ of 2%. The blue-shift of the emission and changes in Φ_PL_ in thin films may be attributed to the ICT state sensitivity to the different polarity of the environment as well as different molecular conformations in the solid state. For **dPXZMePydz**, prompt (τ_p_ = 41 ns, 88.5% weighting) and delayed components (τ_d_ = 1.43 μs, 11.5% weighting) were recorded in time-resolved PL decay experiment. Slight Φ_PL_ sensitivity to air and an appreciable delayed component, which decreases upon cooling to 77 K [Figure 6B, τ_p_ = 47.6 ns (89.8%), τ_d_ = 1.58 μs (10.2%)] indicate that TADF is occurring in **dPXZMePydz**. A high *k*_rISC_ value of 3.9 · 10^6^ s^−1^ and triplet state up-conversion yield Φ_rISC_ close to unity was estimated from the thin film photophysics (Dias et al., 2017) (Supplementary Table 3). On the other hand, **dDMACMePydz** exhibited a PL transient dominated by multiple prompt components [τ_p1_ = 10.7 ns (86.9%), and τ_p2_ = 61.2 ns (12.6%)] and only a weak delayed fluorescence signal [τ_d_ = 1.69 μs (0.5 %)] despite the calculated ΔE_ST_ of both compounds being similar (Figure 6C). In the case of **dDMACMePydz**, the higher-lying T_2_ triplet state should be taken into account (Supplementary Figure 22) as higher energy triplet states are believed to play a crucial role in facilitating TADF mechanism (Etherington et al., 2016; Kobayashi et al., 2017; Noda et al., 2019). Indeed, a very high *k*_rISC_ = 1.4 · 10^7^ s^−1^ was estimated in **dDMACMePydz** assuming that ISC and rISC occur predominantly via T_2_ (Tsuchiya et al., 2020). However, rISC is outcompeted by fast internal conversion to T_1_ and a low probability of endothermic up-conversion to T_2_, leading to negligible Φ_rISC_ ≈ 0.01 (Supplementary Table 4). The Δ*E*_ST_s were measured in PMMA films doped with 10 wt% of **dDMACMePydz** and **dPXZMePydz** (Supplementary Figure 21) and the values were found to be 241 nm and 53 meV, respectively. Changing the host material from PMMA to DPEPO (15 wt% doping) resulted in a further reduction of ΔE_ST_ due to the increased polarity of the host, which preferentially stabilizes the ^1^CT state. The ΔE_ST_ values for **dDMACMePydz** and **dPXZMePydz** in DPEPO were found to decrease to 25 nm and 15 meV, respectively.

In summary, the combination of DFT calculations and PL studies indicate that all three compounds exhibit a significant degree of intersystem crossing as a result of their D-A molecular design. S_0−1,2,3_ excitations dominate the absorption spectrum of **dCzMePydz** and have the highest oscillator strengths among the series. We reason about the fast intersystem crossing in **dCzMePydz** based on a combination of theoretical and experimental evidence. According to DFT calculations this compound should show the strongest PL emission since its oscillator strength for S_0−1_ transition is an order of magnitude larger than in **dPXZMePydz** and **dDMACMePydz** compounds. This is also corroborated with the absorption spectra (Figure 5) where the low energy absorption of **dCzMePydz** exhibits the highest molar extinction coefficient values. However, PL experiments show the opposite trend, **dCzMePydz** exhibits negligible luminescence signal and is the poorest emitter among the series. This must be due to higher non-radiative decay in this compound. To explain this, there must be a different excited state deactivation mechanism other than via the singlet channel. DFT calculations provide a plausible compelling explanation that intersystem crossing to a higher energy triplet state T_4_ is the most likely transition due to its close proximity to S1 (Supplementary Figure 20C) and the large exchange integral values due to larger overlap of the corresponding orbitals. RISC is unlikely to occur as it is outcompeted by internal conversion. The fast k_rISC_ = 3.9 · 10^6^ s^−1^ of **dPXZMePydz** is a result of the small Δ*E*_ST_, which leads to efficient and fast delayed emission (<500 ns in toluene). Although a similarly small Δ*E*_ST_ is calculated for **dDMACMePydz**, no delayed fluorescence is observed, likely due to additional non-radiative decay channel similar to the case of **dCzMePydz** (Supplementary Figure 20B).

### Electroluminescence

**Figure 7:**
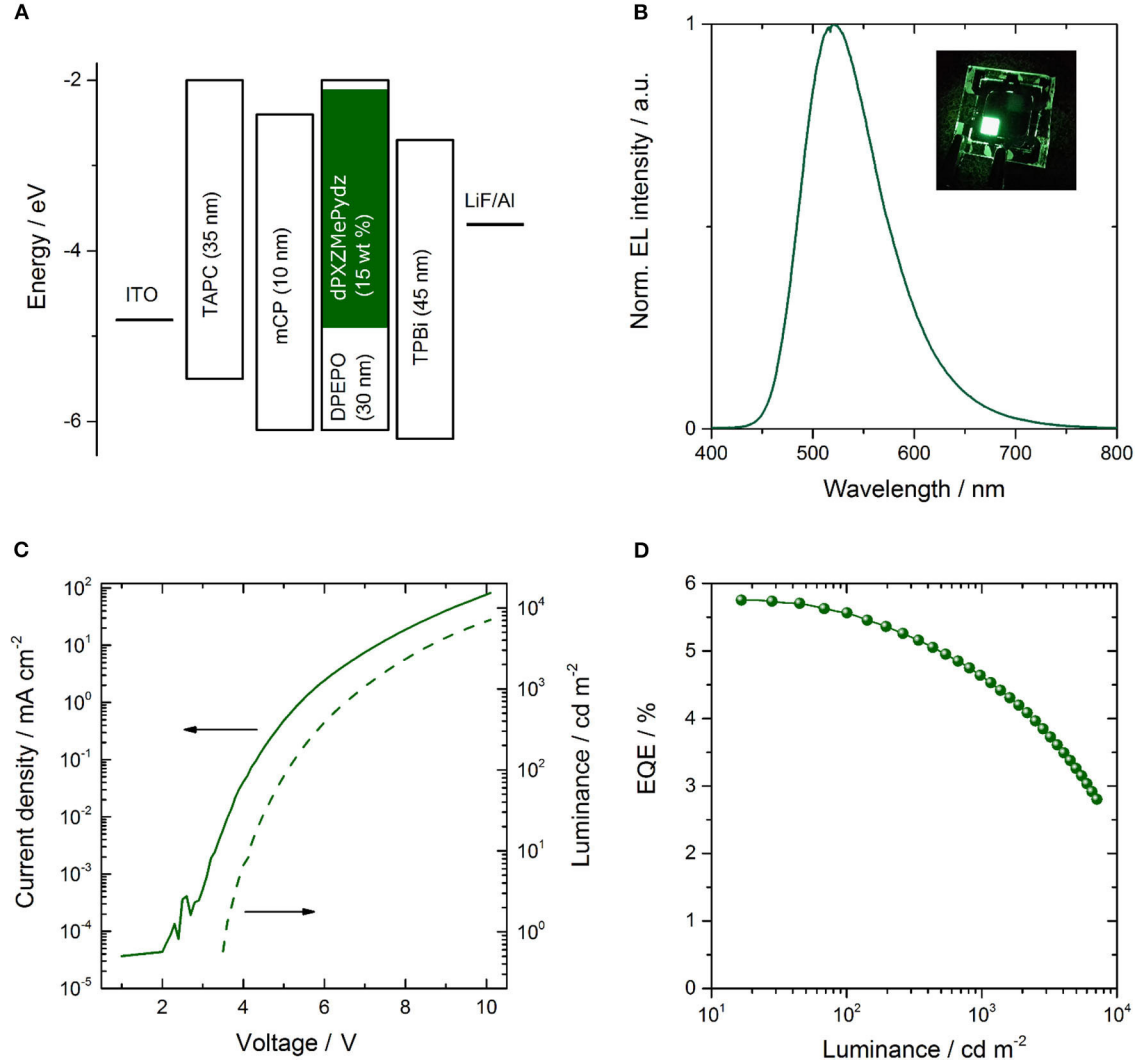
Performance of OLED incorporating **dPXZMePydz** emitter; **(A)** functional layer sequence, layer thicknesses and respective energy levels; **(B)** electroluminescence spectrum, **(C)** current-voltage-luminance characteristics, and **(D)** external quantum efficiency (EQE) vs. luminance.

Due to the presence of TADF in **dPXZMePydz** and this compound having the highest Φ_PL_ of the three compounds, this emitter was incorporated into an OLED structure (Figure 7A). The device structure was: ITO (90 nm)/TAPC (35 nm)/mCP (10 nm)/ **dPXZMePydz**:DPEPO (15 wt%, 30 nm)/TPBi (45 nm)/LiF (1 nm)/Al (100 nm), where 4,4′-cyclohexylidene-bis[*N,N*-bis(4-methylphenyl)benzenamine] (TAPC) is the hole transporting material, 1,3-bis(carbazol-9-yl)benzene (mCP) acts as an electron blocker, and 1,3,5-tris(1-phenyl-1H-benzimidazol-2-yl)benzene (TPBi) is the electron transport layer. The DPEPO host is electron-transporting and has a high triplet energy (E_T_ = 2.9 eV). The mCP interlayer near the expected exciton recombination position also serves to prevent emitter triplet diffusion and quenching. The resulting EL spectrum, current-voltage-luminance and external quantum efficiency (EQE)-luminance characteristics are shown in Figures 7B–D. The OLED exhibits green emission with CIE coordinates of (0.30,0.55) and EQE_max_ of 5.8%. The device performance is summarized in Table 3.

**Table 3:** Summary of dPXZMePydz OLED performance^a^.

**V_**on**_/V^b^**	**λ_EL_/nm**	**CIE**	**EQE/%**^c^				**PE/lm W^**−1**^^d^**
			**Max**	**100 cd m^**−1**^**	**500 cd m^**−1**^**	**5,000 cd m^**−1**^**	
3.5 ± 0.1	520	(0.30, 0.55)	5.8 ± 0.4	5.6 ± 0.1	5.0 ± 0.1	3.2 ± 0.1	10.8 ± 0.3

a *Averaged over four devices produced in the same evaporation batch*.

b *Defined as the voltage at 1 cd m^−2^ brightness*.

c *Calculated assuming Lambertian emission*.

d *At 100 cd m^−2^*.

**Figure 8:**
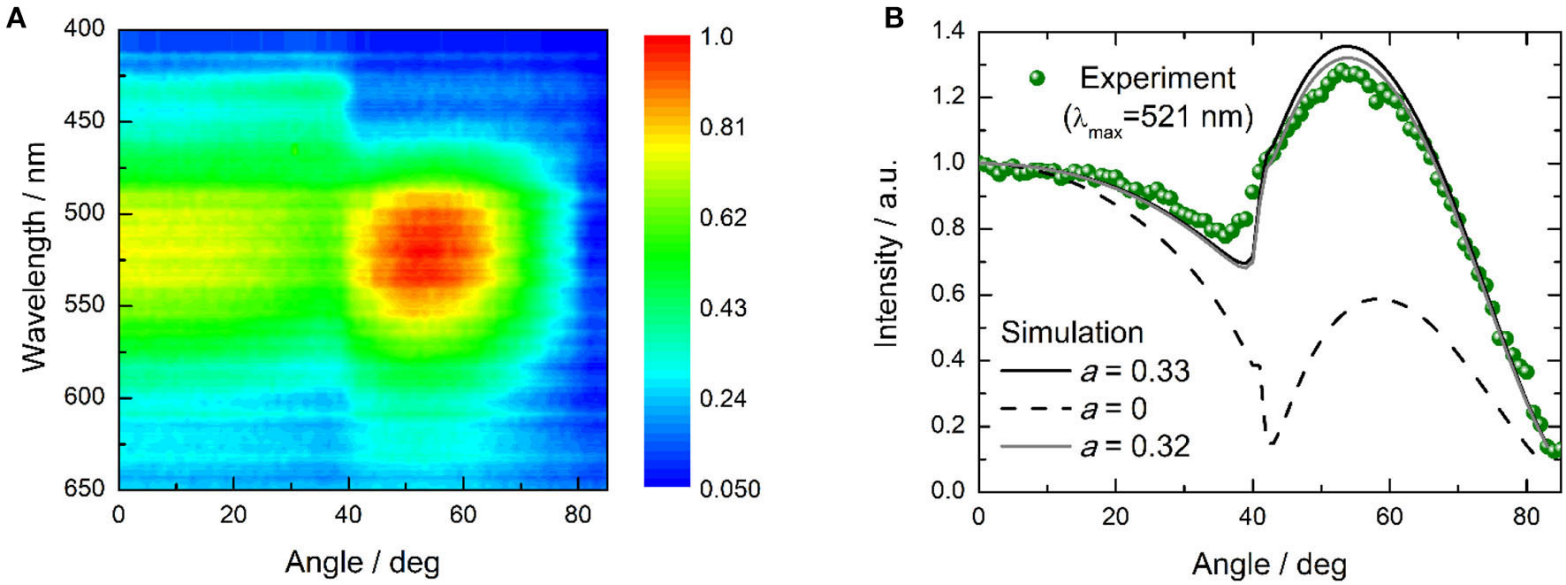
Polarized angular emission spectroscopy characterization of **dPXZMePydz:**DPEPO (15 wt%) film; **(A)** angular dependence of the photoluminescence spectrum (λ_exc_ = 365 nm); **(B)** angular dependence of emission intensity at peak emission wavelength (λ = 521 nm, green disks), and simulated angular dependence for the case of horizontal (*a* = 0, black dashed line) and isotropic (*a* = 0.33, black solid line) emitter orientation as well as for the closest match to the experimental data which was achieved with *a* = 0.32 (gray solid line).

The OLED performance is higher than the theoretical spin statistic limit for a fluorescent OLED. Thus, triplet harvesting clearly occurs during electroluminescence. Assuming that 100% triplets are harvested, the outcoupling efficiency is 0.2–0.3, and taking into account Φ_PL_ of the **dPXZMePydz:**DPEPO (15 wt%) film, the expected EQE of the OLED is 2.6–3.9%, indicating that the recorded EQEs are higher than would be expected even for effective triplet harvesting by TADF. One possibility to explain this is a light outcoupling enhancement due to a deviation from an isotropic emitter orientation to a more horizontal orientation (Liehm et al., 2012; Kim and Kim, 2018). To quantify the effect of the emitter orientation in **dPXZMePydz:**DPEPO films, angular polarized PL experiments were carried out (Figure 8A). The experimental data were then fitted to the optical model with the orientation factor *a* as the fitting parameter as described in Ref. (Graf et al., 2014). The isotropic case is represented by *a* = 0.33 and fully horizontal alignment results in *a* = 0. However, the actual average emitter orientation was found to be very close to the isotropic case (*a* = 0.32, Figure 8B), thus ruling out the explanation of a preferential orientation of the transition dipole moment. Weak microcavity effects could be partially responsible for the higher than expected EQE. A further contribution might arise from differences between the PL and EL processes. In particular, in EL charges could trap on the emitter, leading to direct excitation of the emitter. By contrast, in PL some of the excitation light may be absorbed by the DPEPO host and if energy transfer to the emitter is not perfectly efficient, this would lead to a loss in PL that is not present in EL.

## Conclusions

Herein, pyridazine was explored within three newly synthesized D-A compounds with varying electron-donating strength. Photophysical and DFT calculations revealed strong intersystem crossing occurring in these purely organic compounds. Compounds **dCzMePydz** and **dDMACMePydz**, comprising carbazole and acridine donors, respectively, were poorly emissive and exhibited pure fluorescent emission in the blue and blue-green spectral regions. On the other hand, combining Pydz with phenoxazine resulted in emitter **dPXZMePydz** with moderate green luminescence with Φ_PL_ of 8.5% and a fast delayed emission lifetime of 470 ns in toluene. Experiments in aerated and degassed solutions confirmed TADF being the mechanism responsible for the observed delayed emission. Upon deposition of a thin film containing **dPXZMePydz**, Φ_PL_ increased to 10.9% with an estimated high reverse intersystem crossing rate *k*_rISC_ = 3.9 · 10^6^ s^−1^ and a small singlet-triplet splitting value Δ*E*_ST_ = 86 meV, which was corroborated by TDA-DFT calculations. Theoretical calculations also revealed low energy intermediate triplet states lying in the vicinity of S_1_ and T_1_ in **dCzMePydz** and **dDMACMePydz** compounds. Finally, OLED comprising **dPXZMePydz** in DPEPO host at an optimal concentration of 15 wt% were fabricated via thermal evaporation in vacuum. These OLEDs demonstrated EQE_max_ over 5.8%, confirming efficient triplet utilization in the device via TADF_._ Our results suggest that some pyridazine-containing molecules are of interest as an approach to TADF molecules with high rates of rISC.

## Data Availability Statement

The research data supporting this publication can be accessed at https://doi.org/10.17630/3f2695c7-e6d5-4e11-a0b1-dcf1a54d4bb5.

## Author Contributions

SK co-wrote the manuscript, undertook some of the photophysical measurements, undertook some of the analysis of the data, and fabricated all of the devices. TM co-wrote the manuscript, conducted the DFT calculations, and undertook some of the analysis of the data. SD co-wrote the manuscript, undertook some of the photophysical measurements, and analysis of the data. GC synthesized the compounds, conducted the electrochemical measurements, and some of the photophysical measurements. EA and CK co-wrote the manuscript and undertook some of the orientation measurements. DC co-wrote the manuscript and solved the X-ray structures. AS supervised DC and funded the acquisition of the XRD equipment used in this study. MG co-wrote the manuscript, undertook some of the analysis of the orientation measurements, and supervised EA and CK. IDWS co-managed the project, co-wrote the manuscript, undertook some of the analysis of the data, supervised SK, and co-supervised SD. EZ-C conceived of the molecular design, co-managed the project, co-wrote the manuscript, undertook some of the analysis of the data and supervised TM, GC, and co-supervised SD. All authors contributed to the article and approved the submitted version.

## Conflict of Interest

The authors declare that the research was conducted in the absence of any commercial or financial relationships that could be construed as a potential conflict of interest.
