# Supplementary material for: Fast Delayed Emission in New Pyridazine-Based Compounds
Source: Front Chem. 2021 Jan 7;8:572862. doi: 10.3389/fchem.2020.572862 (PMC7817954; doi:10.3389/fchem.2020.572862)
Supplement: Supplementary file 1 [file Data_Sheet_1.PDF]

Fast Delayed Emission in New Pyridazine-based Compounds

Simonas Krotkus,[a,c] Tomas Matulaitis,[b] Stefan Diesing,[a,b] Graeme Copley,[b,d] Emily Archer,[a] Changmin Keum,[a] David B. Cordes,[b] Alexandra M. Z. Slawin,[b] Malte C. Gather,[a] Eli Zysman-Colman[b] and Ifor D.W. Samuel*[a]*

[a] Organic Semiconductor Centre, SUPA, School of Physics and Astronomy, University of St Andrews, North Haugh, St Andrews, Fife, KY16 9SS, UK

[b] Organic Semiconductor Centre, EaStCHEM School of Chemistry, University of St Andrews, Fife, KY16 9ST, UK

[c] Present address: AIXTRON SE, Dornkaulstrasse 2, 52134, Herzogenrath, Germany

[d] Present address: 930 North University Avenue, Ann Arbor, MI 48109-1055

Supporting Information

Table of contents

General synthetic procedures 3
Synthesis 3
^{1}H and ^{13}C spectra 7
HRMS spectra 12
HPLC traces 14
Elemental analysis 17
X-ray crystallography 20
Theoretical calculations 21
Photophysical measurements 25
Electrochemistry measurements 29
OLED fabrication and characterization 30
Cartesian coordinates 31
References 36

General synthetic procedures

Commercial chemicals were used without further purification. 3-Iodo-6-methylpyridazine was prepared from 3-chloro-6-methylpyridazine following a previously reported procedure (yield 92 %).[1] All reactions were performed using standard Schlenk techniques under nitrogen atmosphere with dry solvents. Column chromatography was performed using silica gel (Silia-P from Silicycle, 60 Å, 40 to 63 μm). Analytical thin layer chromatography was performed with silica plates with polymer (250 μm with indicator F-254), and compounds were visualized under UV light. ^{1}H and ^{13}C solution-phase NMR spectra were recorded on a Bruker Avance spectrometer operating at 11.7 T (Larmor frequencies of 400 and 100 MHz, respectively) in chloroform-*d* ($CDCl_3$) or CD_2Cl_2 solvent. The following abbreviations have been used for multiplicity assignments: "s" for singlet, "d" for doublet, "t" for triplet, "m" for multiplet. High-resolution mass spectra of all compounds were recorded at the Engineering and Physical Sciences Research Council UK National Mass Spectrometry Facility at Swansea University on a Fourier transform mass spectrometer (FTMS), instrument LTQ Orbitrap. Elemental analysis was done by Mr. Stephen Boyer, London Metropolitan University. High-performance liquid chromatography (HPLC) analysis was conducted on a Shimadzu Prominence Modular HPLC system. HPLC traces were measured using an ACE Excel 2 C18 analytical column.

Synthesis

3-(3,5-dibromophenyl)-6-methylpyridazine (MePydz)

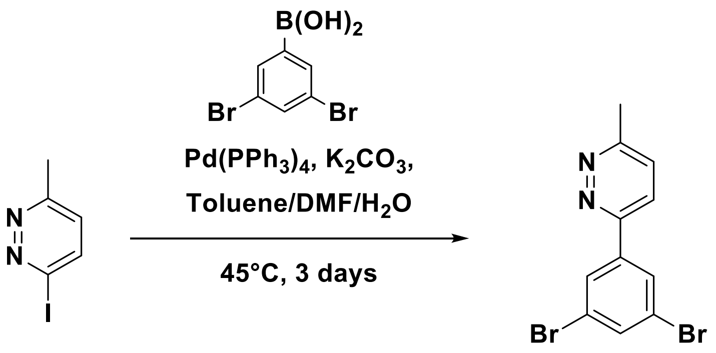

To an oven dried Schlenk flask under nitrogen were added 3-iodo-6-methylpyridazine (2 g, 9.1 mmol, 1.0 equiv.) and (3,5-dibromophenyl)boronic acid (3.31 g, 11.8 mmol, 1.3 equiv.). A mixture of toluene, dimethylformamide

and water (20 mL, 4/1/1 vol. ratio) was added and the mixture was purged with nitrogen for 20 min. After addition of tetrakis(triphenylphosphine)palladium(0) (1.05 g, 0.9 mmol, 0.1 equiv.) and potassium carbonate (3.77 g, 27.2 mmol, 3 equiv.) the reaction tube was quickly sealed, and the contents were stirred while being heated at 45 °C for 72 h. The reaction mixture was then filtered through Celite, and the solvent was evaporated under reduced pressure and then dissolved in dichloromethane (100 mL). The organic phase was washed with water and brine and was dried with Na_2SO_4. After the solvent was removed under reduced pressure, a crude product was obtained and purified by column chromatography on silica gel, eluting with ethyl acetate and hexane (1:2, v/v). **Yield**: 2.44 g (82 %) off-white powder. **R_f**: 0.65 (hexane : dichloromethane = 1:1 on silica gel). **^{1}H NMR**: 8.16 (d, J = 1.8 Hz, 2H), 7.76 (t, J = 1.8 Hz, 1H), 7.71 (d, J = 8.7 Hz, 1H), 7.42 (d, J = 8.7 Hz, 1H), 2.78 (s, 3H). **^{13}C NMR**: 159.61, 154.67, 139.80, 135.08, 128.66, 127.47, 123.82, 123.62, 22.15.

9,9'-(5-(6-methylpyridazin-3-yl)-1,3-phenylene)bis(9H-carbazole) (dCzMePydz)

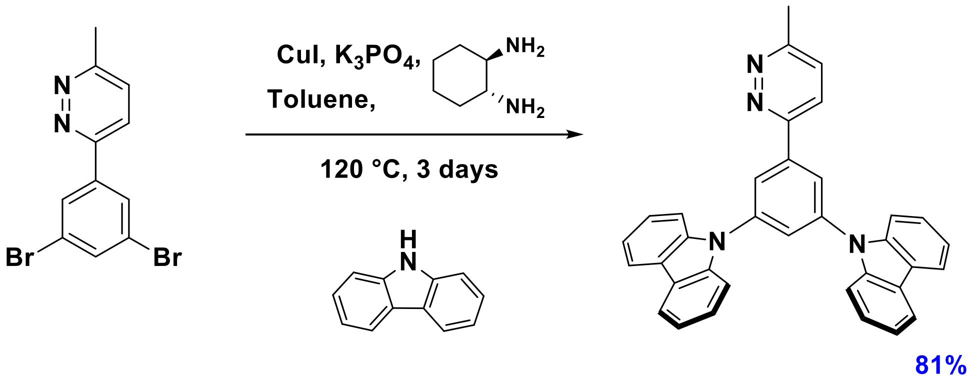

The synthesis was adapted from the literature.[2] To a mixture of CuI (46.75 mg, 0.24 mmol, 0.2 equiv.), K_3PO_4 (647 mg, 3 mmol, 2.48 equiv.) and carbazole (612 mg, 3.66 mmol, 3.02 equiv.) in toluene (20 mL) in a Schlenk tube were added 3-(3,5-dibromophenyl)-6-methylpyridazine (400 mg, 1.21 mmol, 1 equiv.) and (±)-*trans*-1,2-diaminocyclohexane (55.1 mg, 0.48 mmol, 0.4 equiv.) under nitrogen. The reaction tube was quickly sealed, and the contents were stirred at 110 °C for 72 h. The reaction mixture was then filtered through Celite, and the solvent was evaporated under reduced pressure and then dissolved in dichloromethane (100 mL). The organic phase was washed with water and brine and was dried with Na_2SO_4. After the solvent was removed under reduced pressure,

a crude product was obtained and purified by column chromatography on silica gel, eluting with ethyl acetate and hexane (1:4, v/v). **Yield**: 494 mg (81 %) off-white powder. **R$_f$**: 0.6 (hexane : EtOAc = 1:1 on silica gel) **^{1}H NMR**: 8.43 (d, 2H, J = 1.79 Hz), 8.19 (d, 4H, J = 7.76 Hz), 7.97 (t, 1H, J = 1.71 Hz), 7.88 (d, 1H, J = 8.76 Hz), 7.65 (d, 4H, J = 8.24 Hz), 7.46-7.49 (m, 5H), 7.33 (t, 4H, J = 7.54 Hz), 2.74 (s, 3H). **^{13}C NMR**: 159.5, 155.8, 140.5, 140.2, 140.1, 127.5, 126.3, 126.0, 124.0, 124.0, 123.7, 120.6, 120.5, 109.8, 22.2. **HRMS [M+H]$^+$ Calculated**: ($C_{35}H_{24}N_4$) 501.2074; **Found:** 501.2067. **Elemental analysis: Calculated** for $C_{35}H_{24}N_4$: C, 83.98; H, 4.83; N, 11.19. **Found**: C, 83.87; H, 4.91; N, 11.07. **HPLC:** 5% H_2O/MeOH, 1.0 mL min^{-1}, 254 nm; tr (99.2 %) = 15.4 min.

10,10'-(5-(6-methylpyridazin-3-yl)-1,3-phenylene)bis(10H-phenoxazine) (dPXZMePydz)

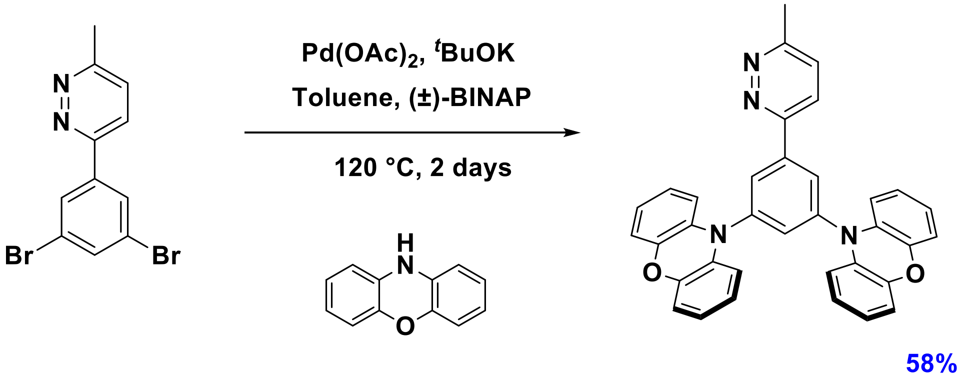

To an oven dried Schlenk flask were added under nitrogen palladium acetate (13.7 mg, 0.06 mmol, 0.1 equiv.) and (±)-BINAP (75.9 mg, 0.12 mmol, 0.2 equiv.). Toluene (10 mL) was added and the mixture was heated to 60 °C for 20 min and then cooled down to room temperature. After addition of 3-(3,5-dibromophenyl)-6-methylpyridazine (200 mg, 0.61 mmol, 1 equiv.) and phenoxazine (560 mg, 3.05 mmol, 5 equiv.) the reaction mixture was heated to 60 °C for 30 min. Potassium *tert*-butoxide (274 mg, 2.44 mmol, 4 equiv.) was then added to the reaction mixture. The reaction tube was quickly sealed, and the contents were stirred while being heated at 110 °C for 48 h. The reaction mixture was then filtered through Celite, and the solvent was evaporated under reduced pressure and then dissolved in dichloromethane (100 mL). The organic phase was washed with water and brine and was dried with Na_2SO_4. After the solvent was removed under reduced pressure, a crude product was

obtained and purified by column chromatography on silica gel, eluting with ethyl acetate and hexane (1:2, v/v). **Yield**: 191 mg (58 %) light yellow powder. **R$_f$**: 0.45 (hexane : EtOAc = 1:1 on silica gel). **^{1}H NMR**: 8.23 (d, 2H, J = 1.87 Hz), 7.80 (d, 1H, J = 8.76 Hz), 7.54 (t, 1H, J = 1.86 Hz), 7.44 (d, 1H, J = 8.79 Hz), 6.74 (dd, 4H, J = 7.75 Hz, J = 1.78 Hz), 6.69 (td, 4H, J = 7.78 Hz, J = 1.73 Hz), 6.11 (dd, 4H, J = 7.79 Hz, J = 1.52 Hz), 2.78 (s, 3H). **^{13}C NMR**: 159.6, 155.1, 143.9, 142.6, 142.5, 135.0, 133.8, 129.6, 127.5, 123.8, 123.4, 121.9, 115.8, 113.3, 22.2. **HRMS [M+H]$^+$ Calculated**: ($C_{35}H_{24}O_2N_4$) 533.1972; **Found:** 533.1964. **Elemental analysis: Calculated** for $C_{35}H_{24}O_2N_4$: C, 78.93; H, 4.54; N, 10.52; O, 6.01. **Found**: C, 79.00; H, 4.65; N, 10.44; O, 5.92. **HPLC:** 5% H_2O/MeOH, 1.0 mL min^{-1}, 254 nm; tr (99.6 %) = 13.6 min.

10,10'-(5-(6-methylpyridazin-3-yl)-1,3-phenylene)bis(9,9-dimethyl-9,10-dihydroacridine) (dDMACMePydz)

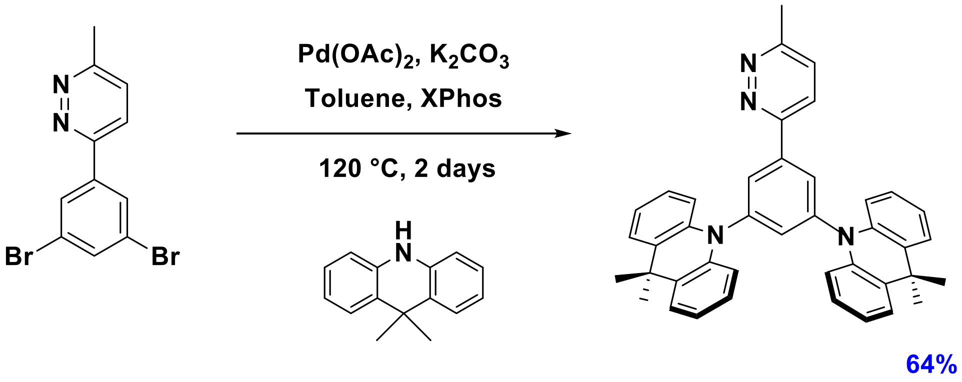

To an oven dried Schlenk flask palladium acetate (27.38 mg, 0.12 mmol, 0.1 equiv.) and (±)-BINAP (75.9 mg, 0.12 mmol, 0.1 equiv.) were added under nitrogen. Toluene (10 mL) was added and the catalyst was heated up to 60 °C for 20 min and cooled down back to room temperature. After addition of 3-(3,5-dibromophenyl)-6-methylpyridazine (400 mg, 1.21 mmol, 1 equiv.) and 9,9-dimethyl-9,10-dihydroacridine (773 mg, 3.66 mmol, 3.02 equiv.) the reaction mixture was heated to 60 °C for 30 min, then potassium *tert*-butoxide (547 mg, 4.87 mmol, 4.02 equiv.) was added. The reaction tube was quickly sealed, and the contents were stirred while being heated at 110 °C for 48 h. The reaction mixture was then filtered through Celite, and the solvent was evaporated under reduced pressure and then dissolved in dichloromethane (100 mL). The organic phase was washed with water and brine and was dried with Na_2SO_4. After the solvent was removed under reduced pressure, a crude

product was obtained and purified by column chromatography on silica gel, eluting with ethyl acetate and hexane (2:1, v/v). **Yield**: 456 mg (64 %) off-white powder. **R$_f$**: 0.55 (hexane : EtOAc = 1:1 on silica gel). **^{1}H NMR**: 8.30 (d, 2H, J = 1.67 Hz), 7.83 (d, 1H, J = 8.79 Hz), 7.52 (t, 1H, J = 0.76 Hz), 7.50 (dd, 4H, J = 6.65 Hz, J = 1.11 Hz), 7.42 (d, 1H, J = 8.80 Hz), 7.05-7.08 (m, 4H), 6.98 (t, 4H, J = 7.36 Hz), 6.54 (d, 4H, J = 8.16 Hz), 2.78 (s, 3H), 1.71 (s, 12H). **^{13}C NMR**: 159.4, 155.5, 144.6, 142.0, 140.5, 136.0, 130.4, 129.9, 127.5, 126.6, 125.4, 123.9, 121.0, 114.1, 36.1, 31.2, 22.1. **HRMS [M+H]$^+$ Calculated**: ($C_{41}H_{36}N_4$) 585.3013; **Found:** 585.3001. **Elemental analysis: Calculated** for $C_{41}H_{36}N_4$: C, 84.21; H, 6.21; N, 9.58. **Found**: C, 84.11; H, 6.31; N, 9.52. **HPLC:** 10% H_2O/MeOH, 1.0 mL min^{-1}, 254 nm; tr (97.6 %) = 11.1 min.

^{1}H and ^{13}C spectra

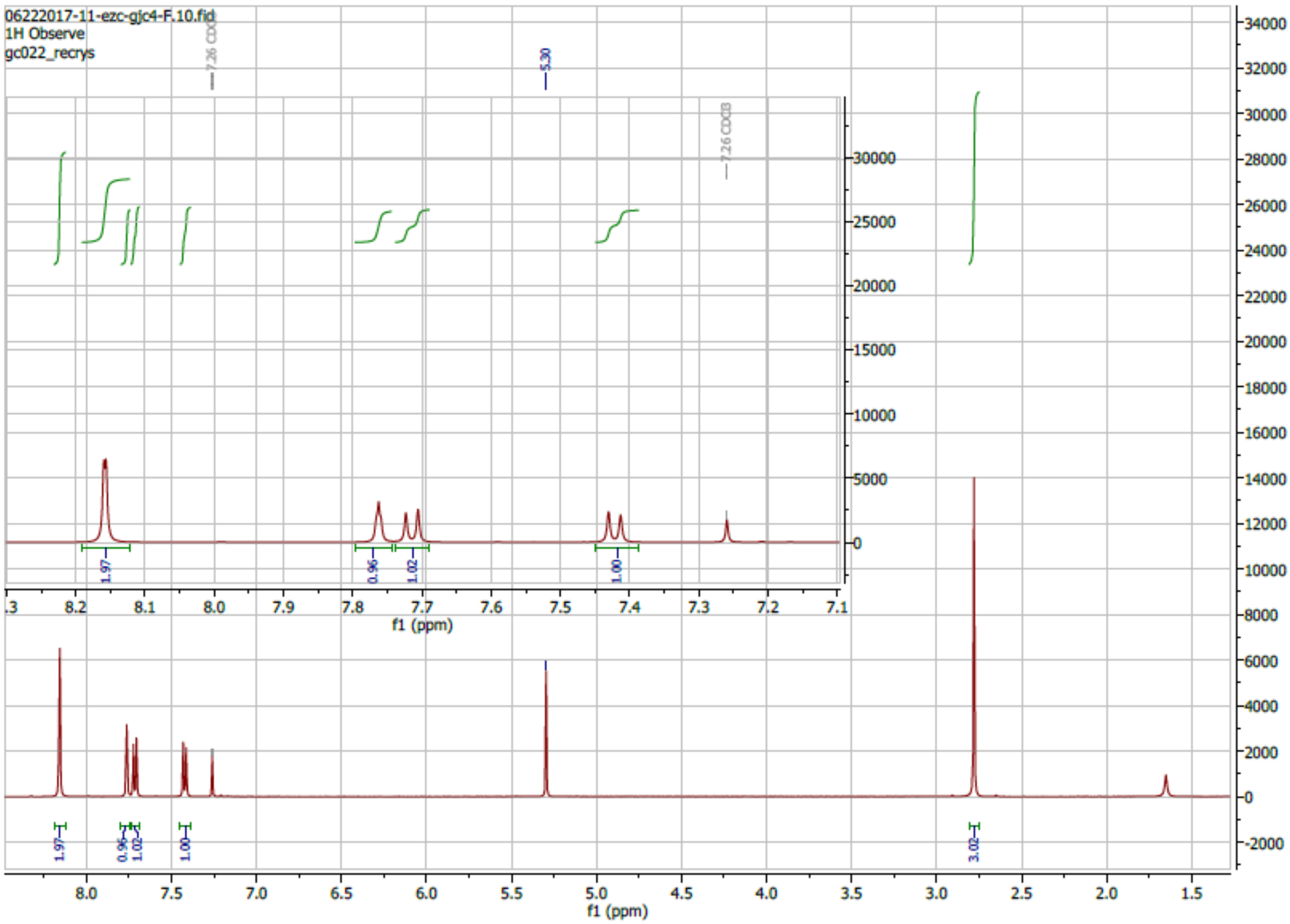

Figure **S1**. ^{1}H NMR spectrum of **MePydz** in $CDCl_3$

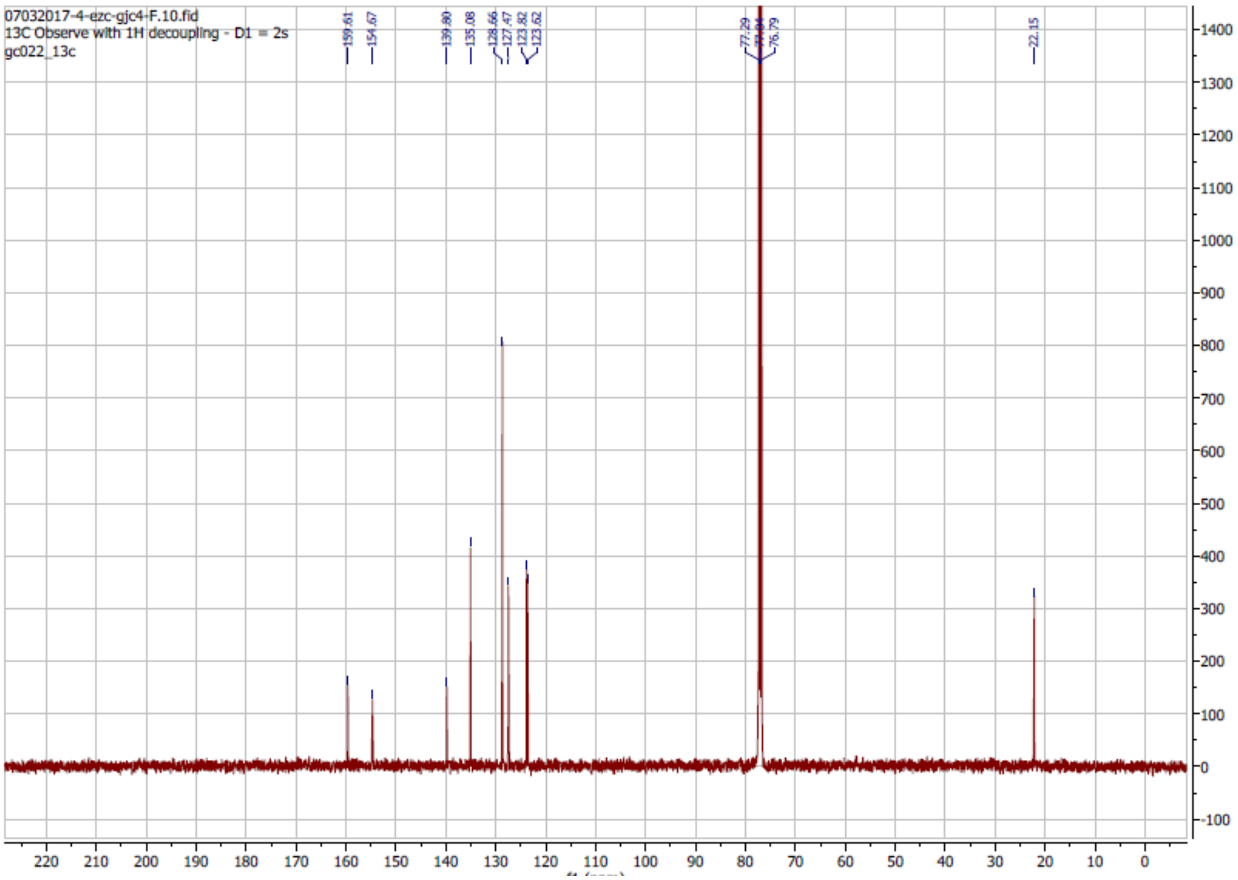

Figure **S2**. ^{13}C NMR spectrum of **MePydz** in $CDCl_3$

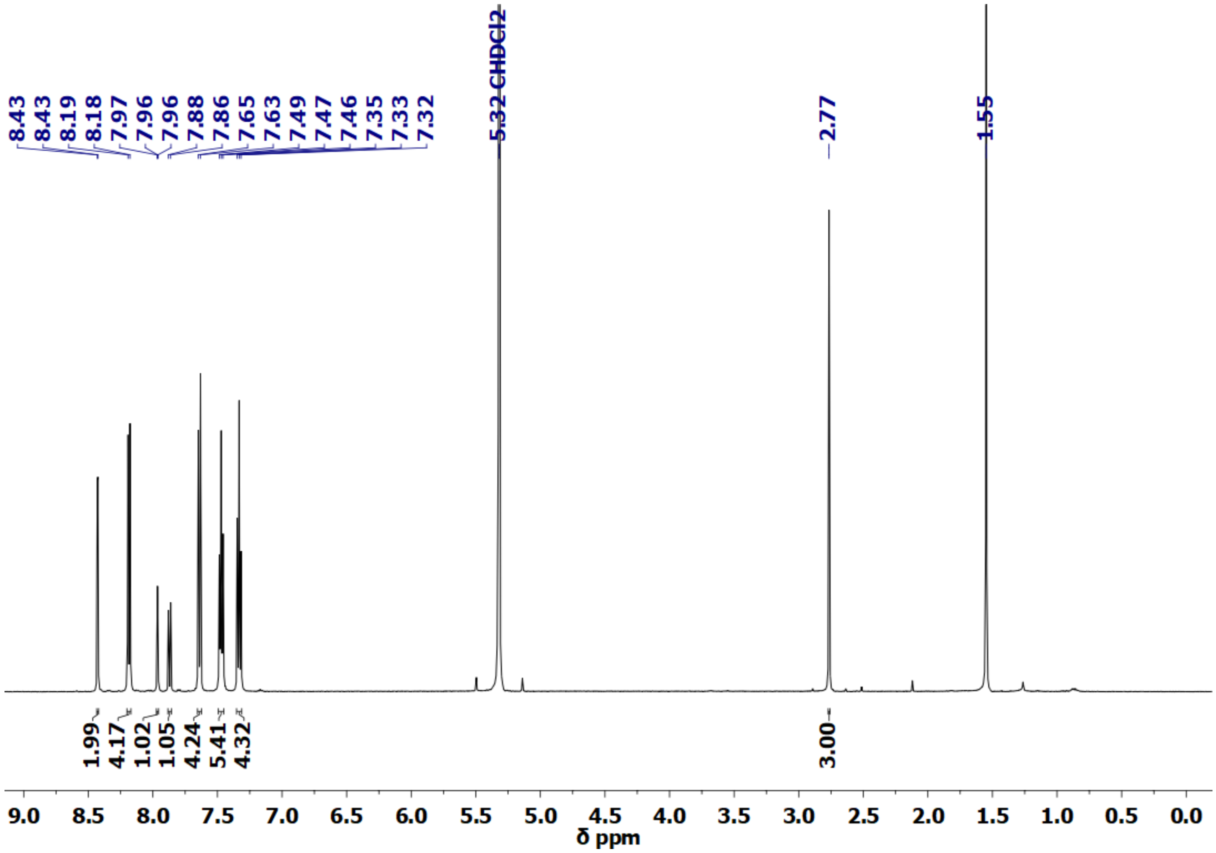

Figure **S3**. ^{1}H NMR spectrum of **dCzMePydz** in CD_2Cl_2

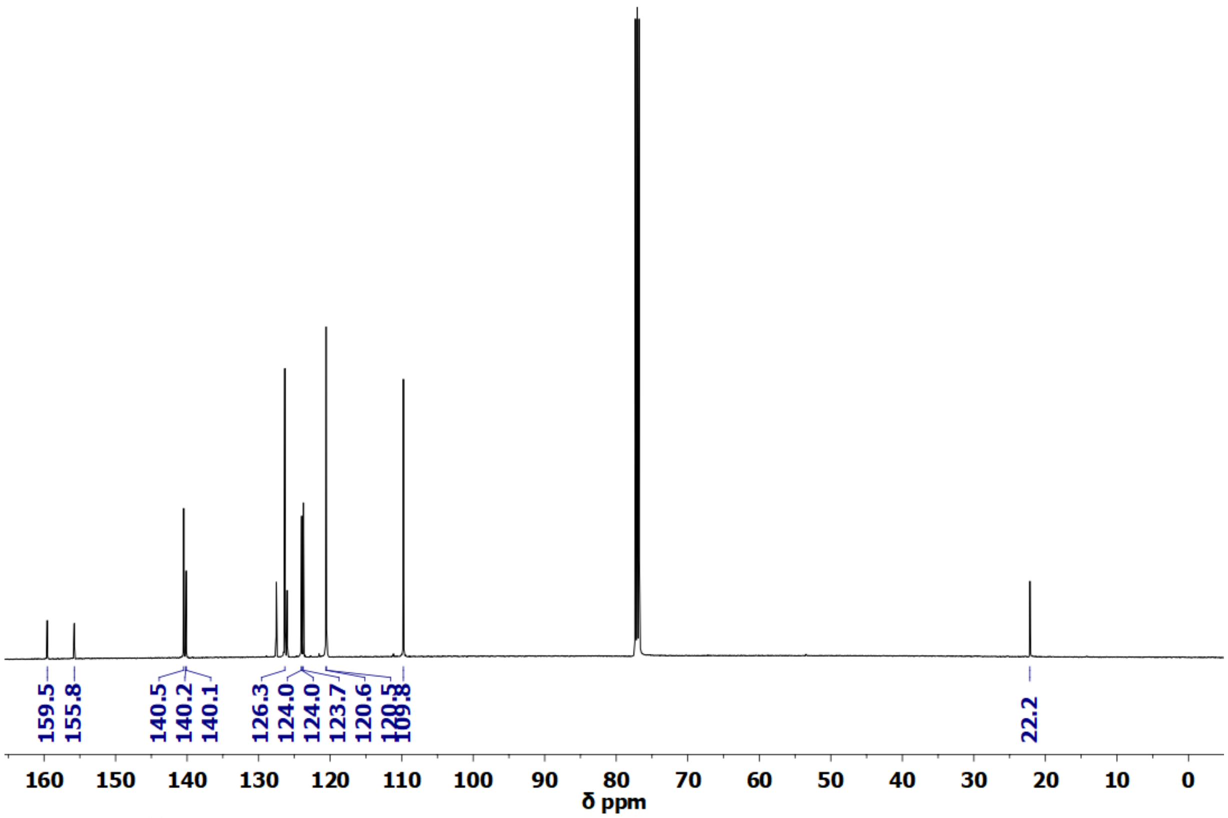

Figure **S4**. ^{13}C NMR spectrum of **dCzMePydz** in $CDCl_3$

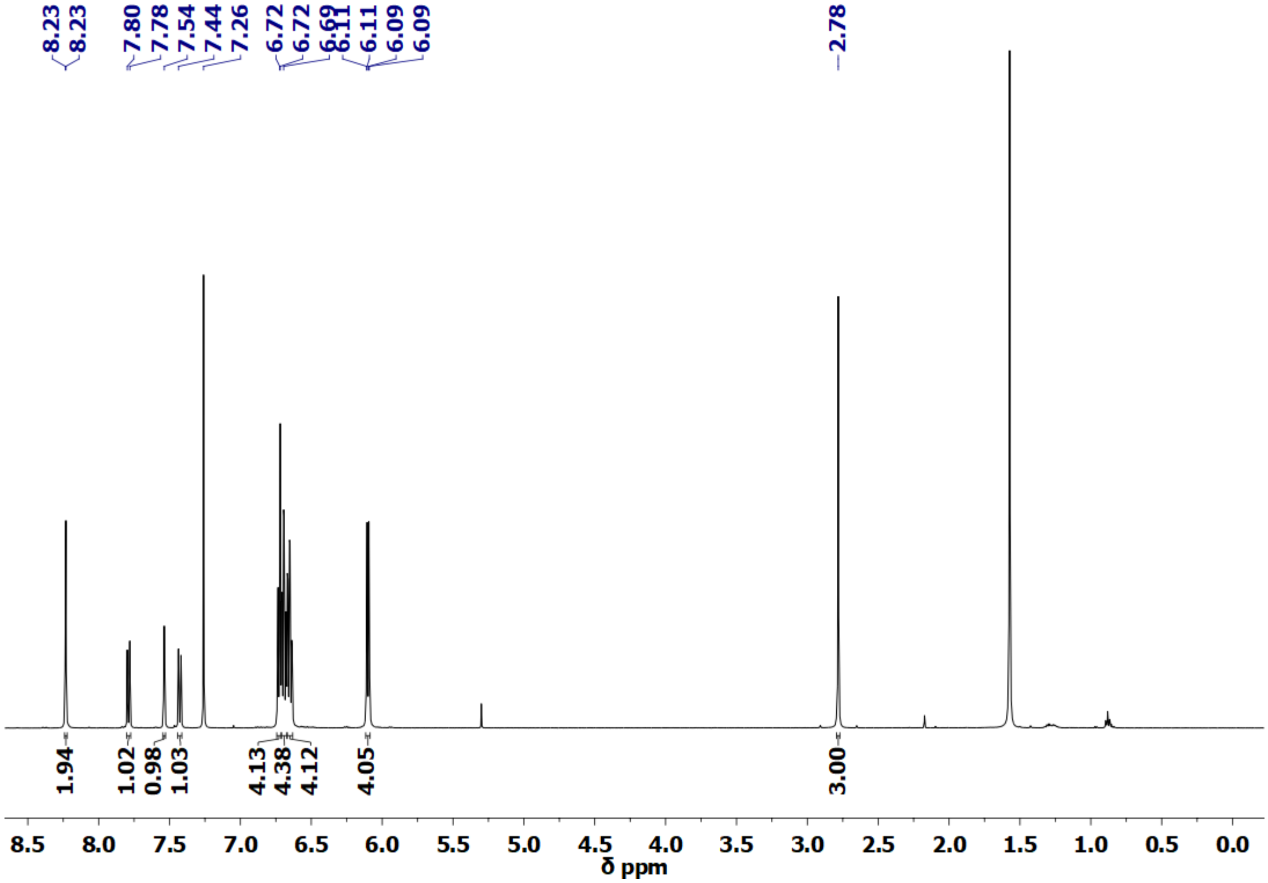

Figure **S5**. ^{1}H NMR spectrum of **dPXZMePydz** in $CDCl_3$

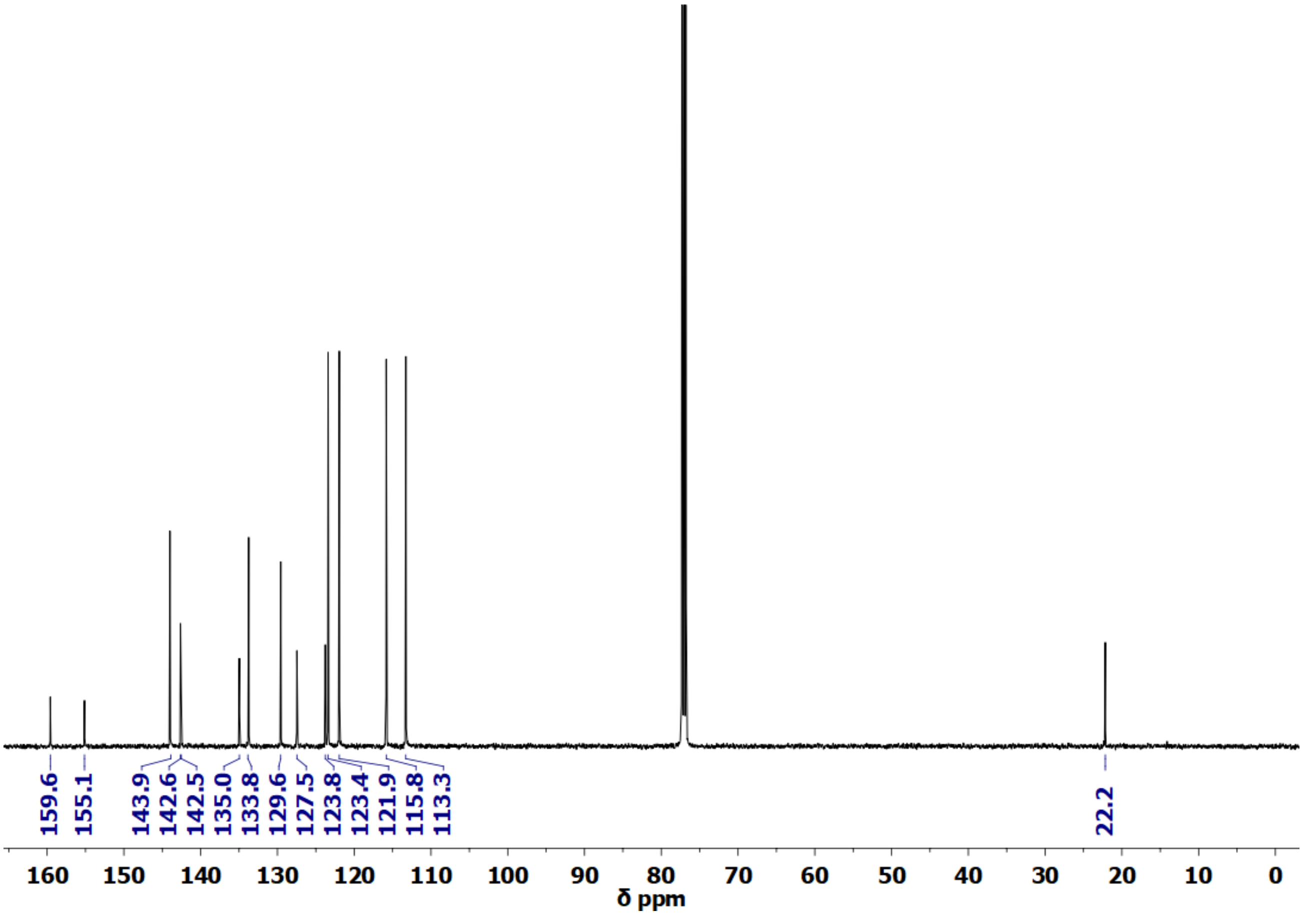

Figure **S6**. ^{13}C NMR spectrum of **dPXZMePydz** in $CDCl_3$

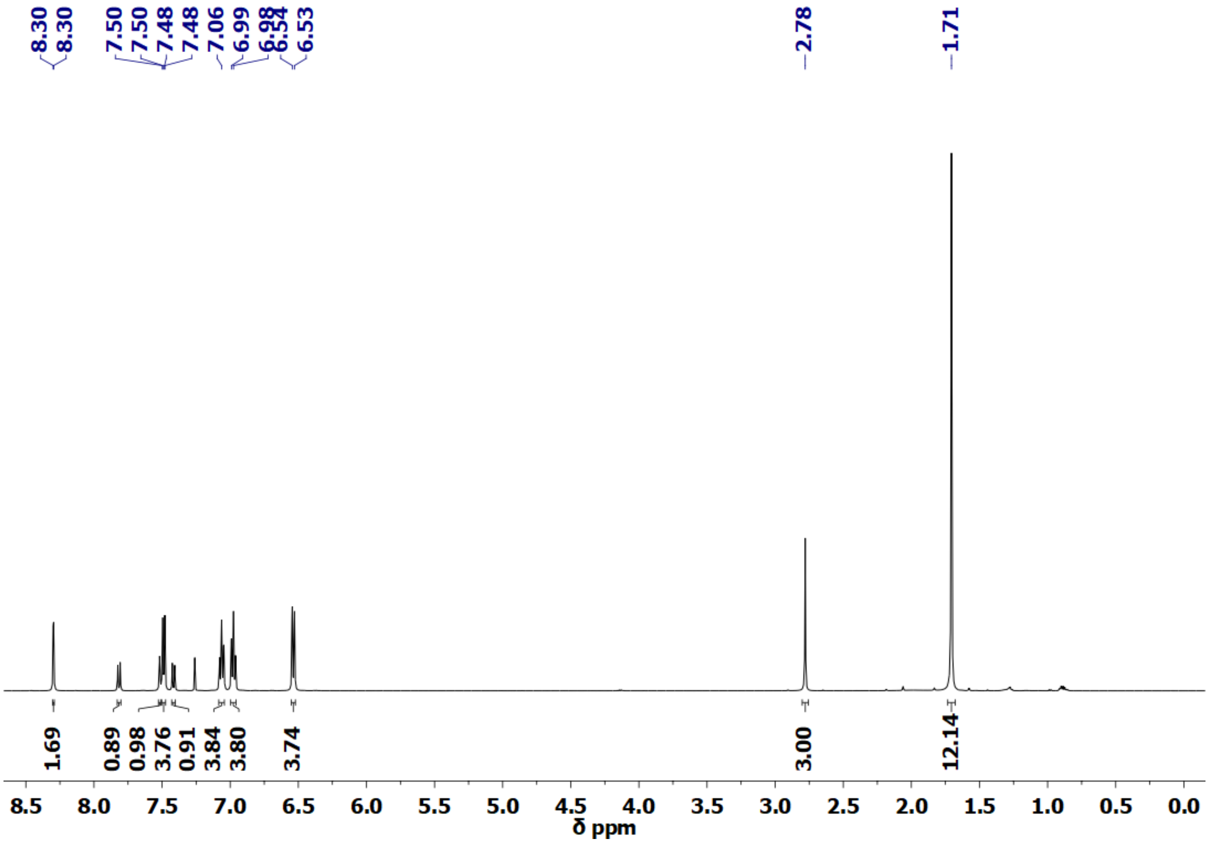

Figure **S7**. ^{1}H NMR spectrum of **dDMACMePydz** in $CDCl_3$

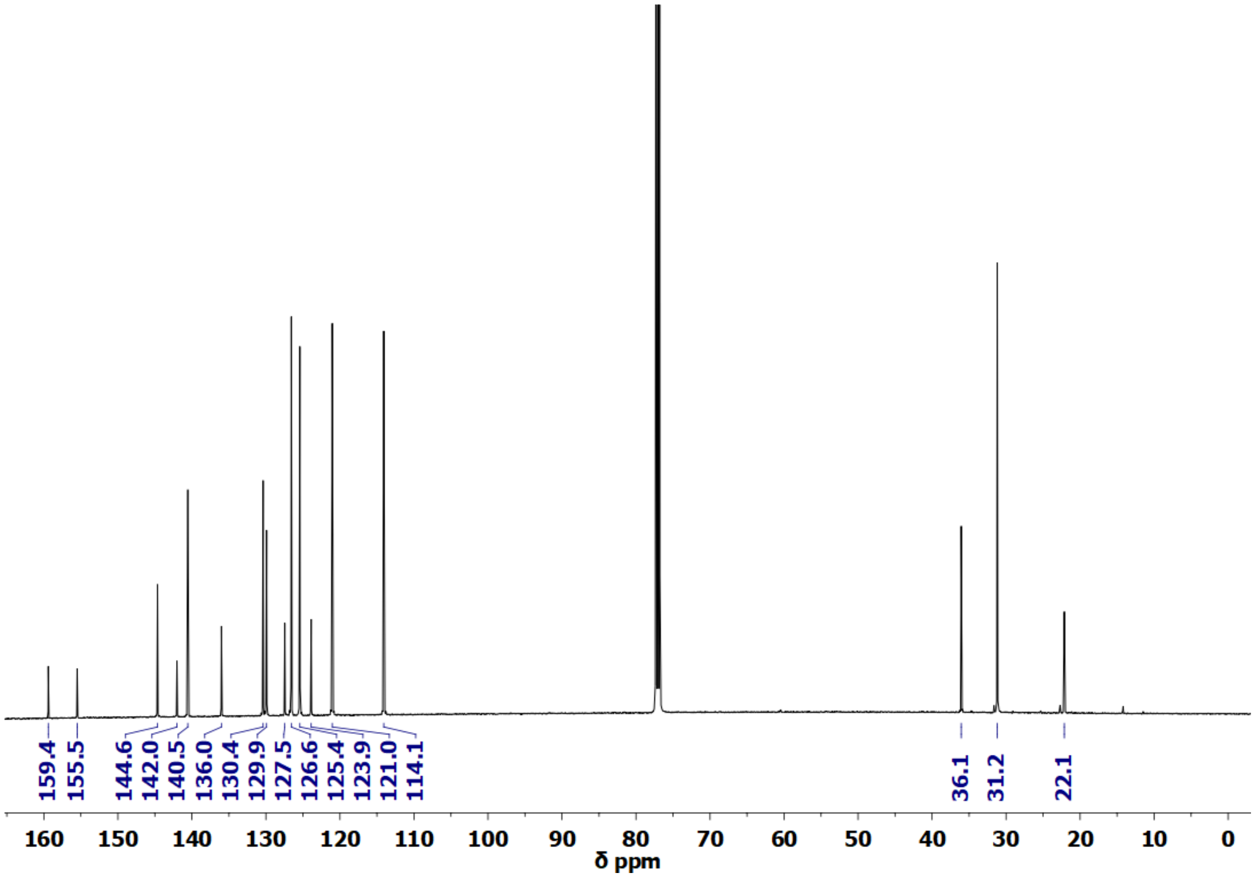

Figure **S8**. ^{13}C NMR spectrum of **dDMACMePydz** in $CDCl_3$

HRMS spectra

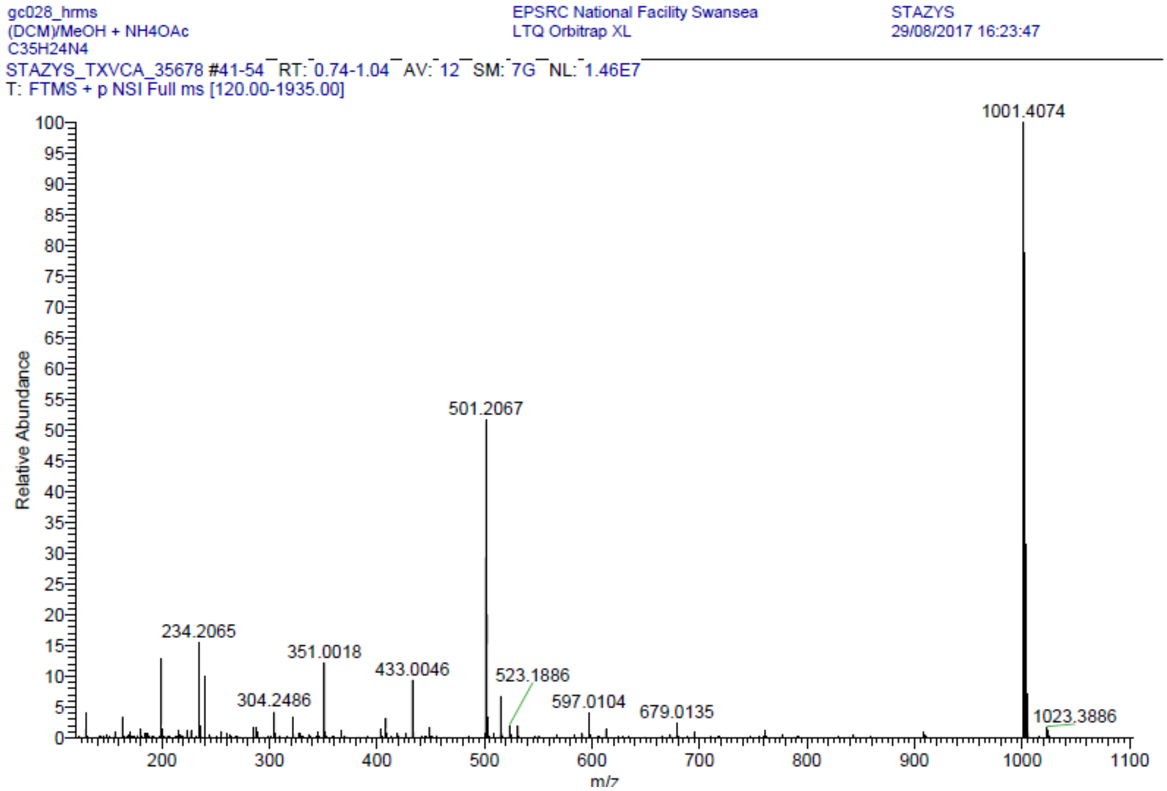

Figure **S9**. HRMS spectrum of **dCzMePydz**

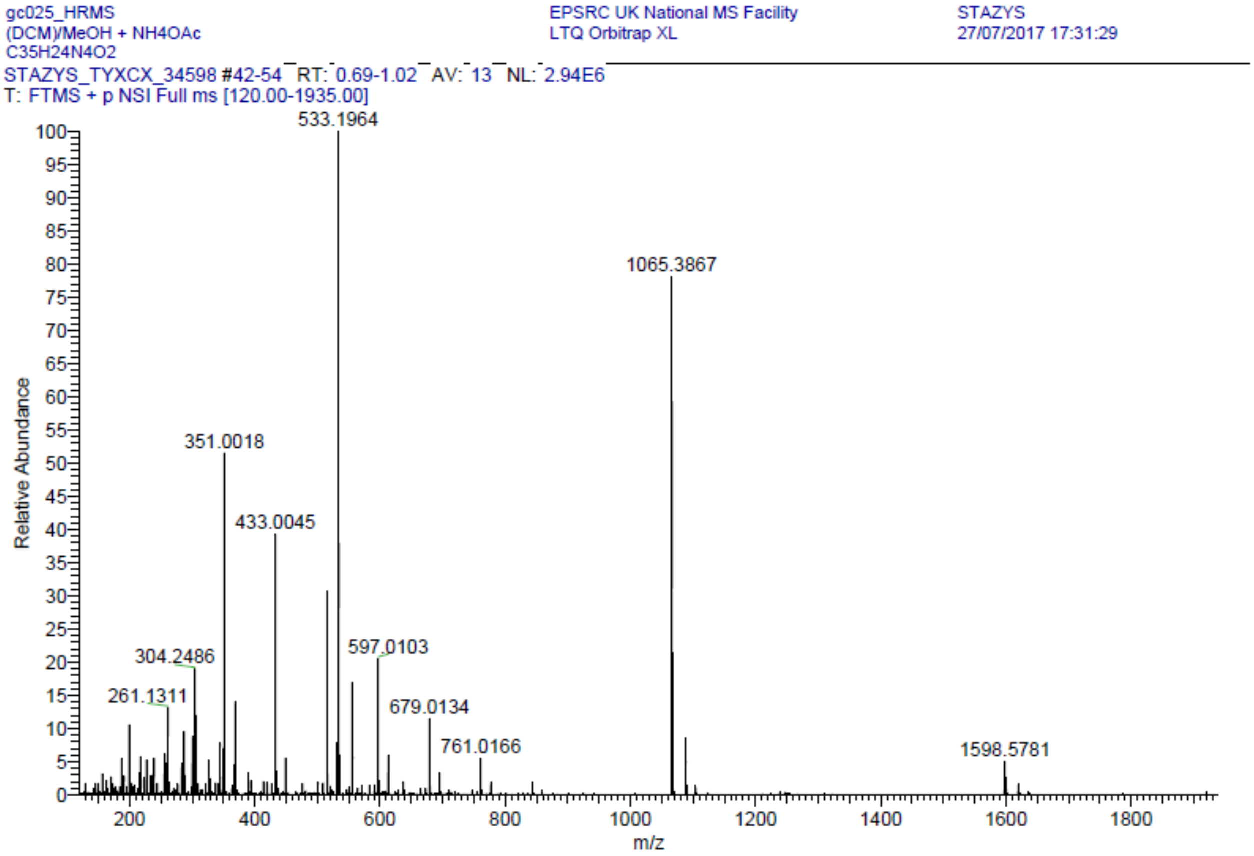

Figure **S10**. HRMS spectrum of **dPXZMePydz**

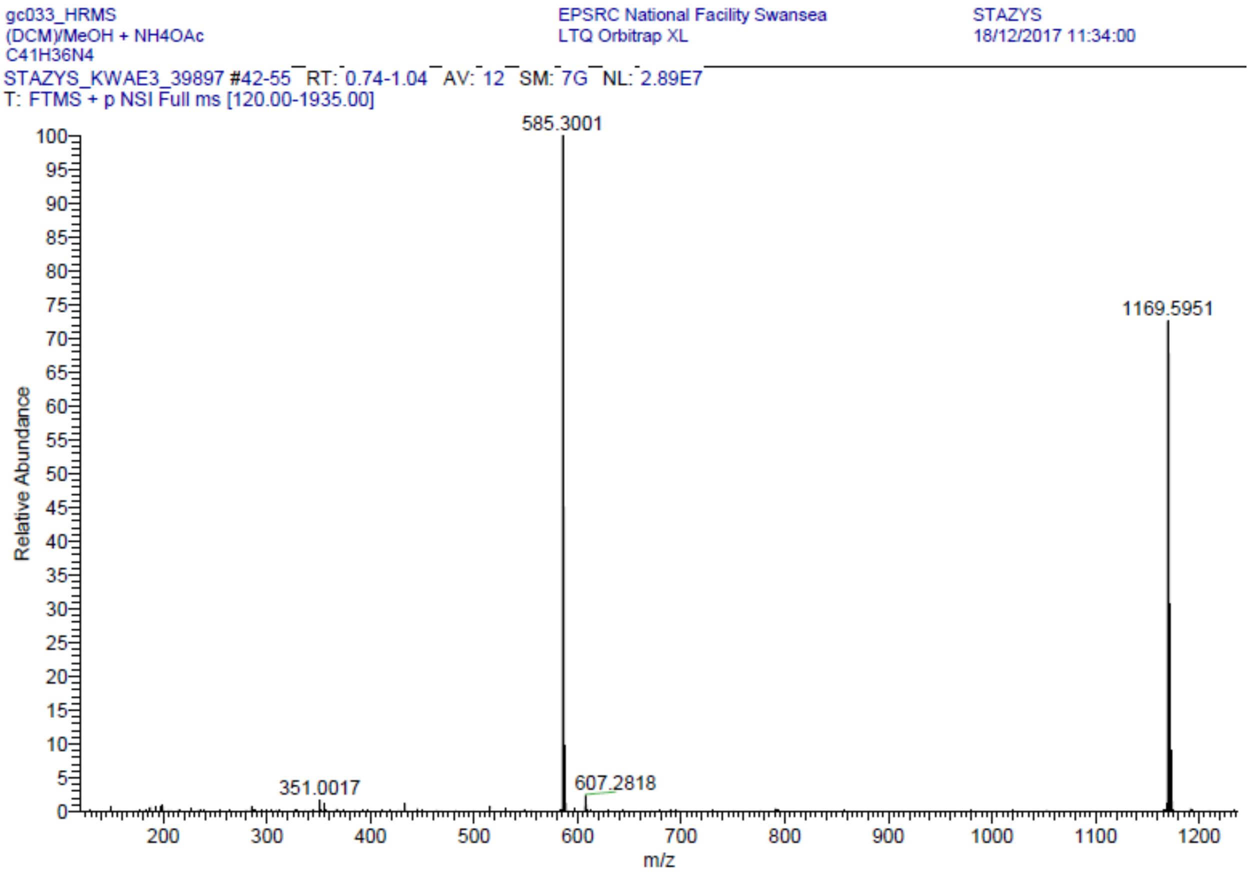

Figure **S11**. HRMS spectrum of **dDMACMePydz**

HPLC traces

17/08/2017 14:24:22 Page 1 / 1

SHIMADZU LabSolutions

Analysis Report

<Sample Information>

Sample Name	: gc028		
Sample ID	: gc028		
Data Filename	: gc028_1.lcd		
Method Filename	: MeOH (95).lcm		
Batch Filename	:		
Vial #	: 1-16	Sample Type	: Unknown
Injection Volume	: 5 uL		
Date Acquired	: 17/08/2017 11:47:11	Acquired by	: ezc-7
Date Processed	: 17/08/2017 12:17:14	Processed by	: ezc-7

<Chromatogram>

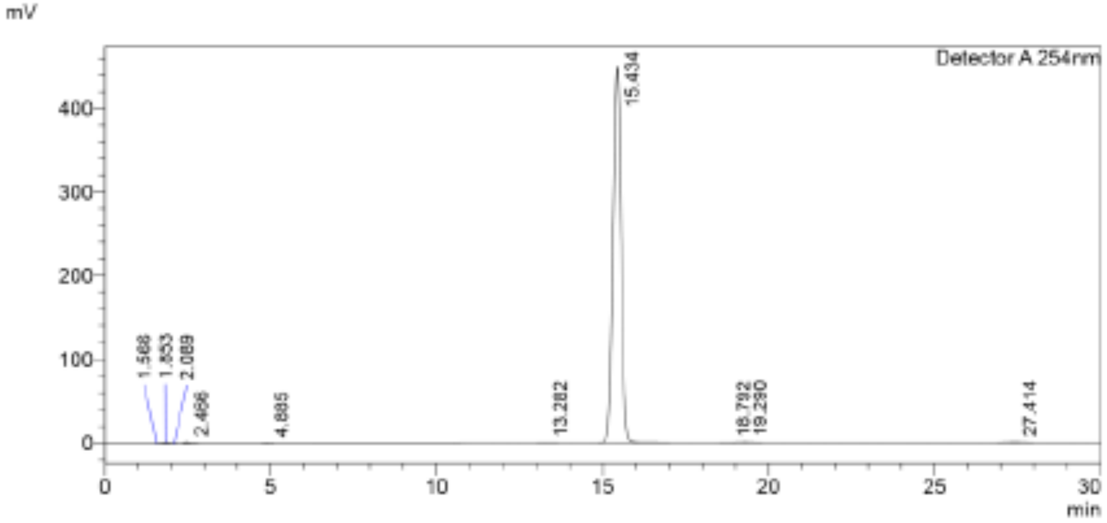

<Peak Table>

Detector A 254nm

Peak#	Ret. Time	Area	Height	Area%	Name
1	1.566	2671	582	0.036	
2	1.853	12045	1213	0.161	
3	2.089	1147	246	0.015	
4	2.466	12082	1472	0.162	
5	4.885	1953	208	0.026	
6	13.282	1907	120	0.025	
7	15.434	7420712	448674	99.213	
8	18.792	1522	85	0.020	
9	19.290	10389	492	0.139	
10	27.414	15182	595	0.203	
Total		7479608	453687	100.000	

Quick Notes Page 1

Figure **S12**. HPLC trace of **dCzMePydz**

16/08/2017 12:56:48 Page 1 / 1

SHIMADZU LabSolutions **Analysis Report**

<Sample Information>

Sample Name	: gc025		
Sample ID	: gc025		
Data Filename	: 1.lcd		
Method Filename	: MeOH (95).lcm		
Batch Filename	:		
Vial #	: 1-16	Sample Type	: Unknown
Injection Volume	: 10 uL		
Date Acquired	: 16/08/2017 12:18:09	Acquired by	: ezc-7
Date Processed	: 16/08/2017 12:48:11	Processed by	: ezc-7

<Chromatogram>

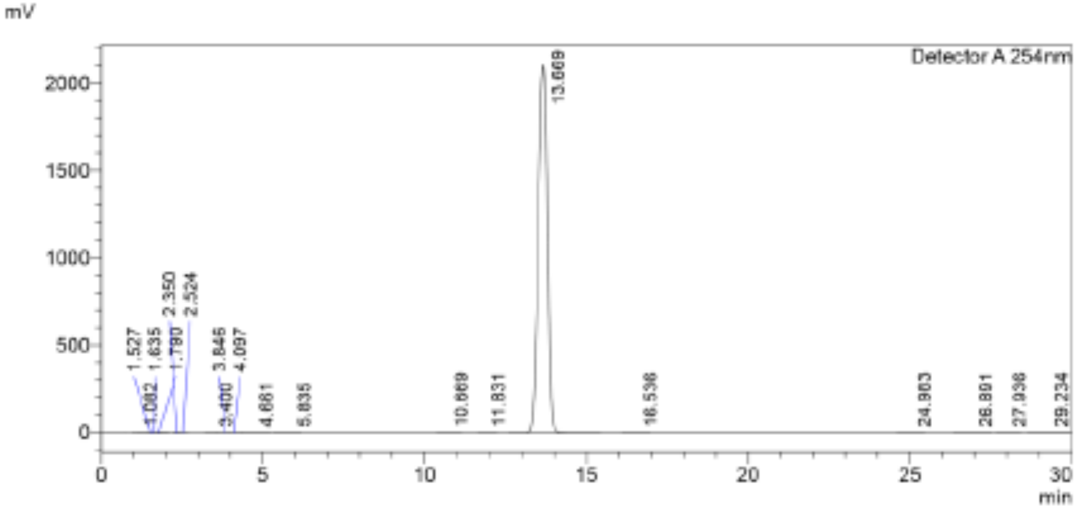

<Peak Table>

Detector A 254nm

Peak#	Ret. Time	Area	Height	Area%	Name
1	1.082	2161	267	0.005	
2	1.527	6239	865	0.016	
3	1.635	3081	763	0.008	
4	1.790	28900	2671	0.072	
5	2.350	1005	97	0.002	
6	2.524	1155	108	0.003	
7	3.400	1508	83	0.004	
8	3.846	5660	415	0.014	
9	4.097	10576	573	0.026	
10	4.661	10083	410	0.025	
11	5.835	8446	441	0.021	
12	10.669	6448	338	0.016	
13	11.831	2333	120	0.006	
14	13.669	40071348	2101272	99.611	
15	16.536	9449	426	0.023	
16	24.983	3569	135	0.009	

Quick Notes Page 1

Figure **S13**. HPLC trace of **dPXZMePydz**

06/12/2017 21:35:39 Page 1 / 1

HPLC Trace Report06Dec2017

<Sample Information>

Sample Name	: gc033_run4		
Sample ID	: gc033		
Method Filename	: MeOH (90).lcm		
Batch Filename	: gc033_061217-02.lcb		
Vial #	: 1-64	Sample Type	: Unknown
Injection Volume	: 15 uL		
Date Acquired	: 06/12/2017 19:21:21	Acquired by	: ezc-7
Date Processed	: 06/12/2017 21:23:01	Processed by	: ezc-7

<Chromatogram>

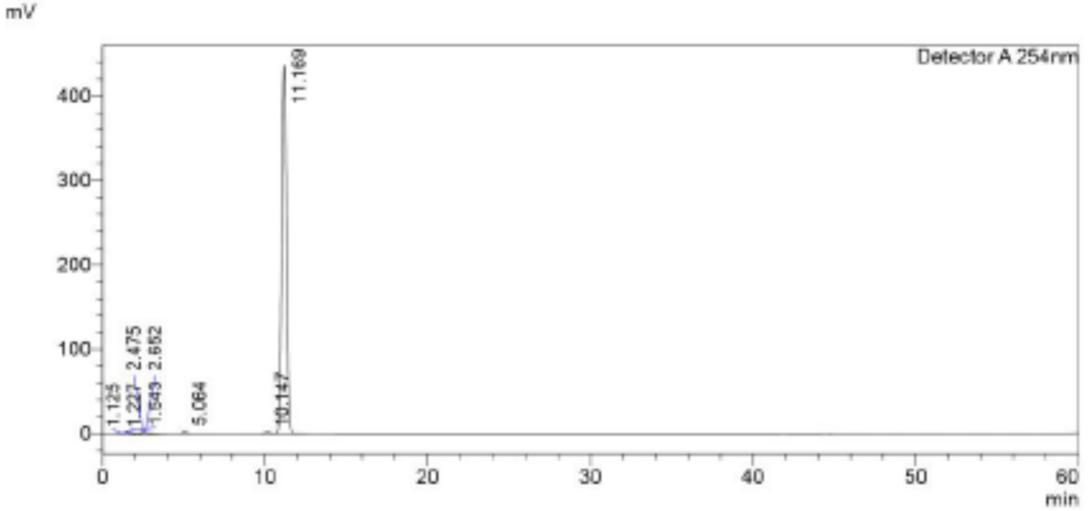

<Peak Table>

Detector A 254nm

Peak#	Ret. Time	Area	Height	Area%	Area/Height	Width at 5% Height
1	1.125	7869	838	0.092	9.395	--
2	1.227	2058	511	0.024	4.025	--
3	1.543	41729	2778	0.490	15.019	0.701
4	2.475	26885	1603	0.316	16.770	--
5	2.652	47206	1645	0.555	28.693	--
6	5.064	36249	2910	0.426	12.458	0.376
7	10.147	34824	1849	0.409	18.833	0.579
8	11.169	8311442	435456	97.687	19.087	0.594
Total		8508260	447591	100.000		

Quick Notes Page 1

Figure **S14**. HPLC trace of **dDMACMePydz**

Elemental analysis

0.4% for solid.
0.7% for oil.

LONDON
metropolitan
university

Elemental Analysis Service

Please send completed form and samples to:

Stephen Boyer
School of Human Sciences
Science Centre
London Metropolitan University
29 Hornsey Road
London N7 7DD

Telephone: 020 7133 3605
Fax: 020 7133 2577
Email: s.boyer@londonmet.ac.uk

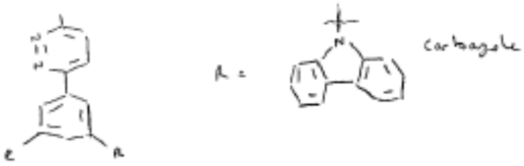

Sample submitted by: Graeme Copley	
Address: **EZC group, School of Chemistry, University of St Andrews, North Haugh,St Andrews, Fife, KY16 9ST**	
Telephone: 07931680962	Email: gjc4@st-andrews.ac.uk
Date Submitted: 23/08/2017	

Please submit ca. 5 mg of sample.

Sample Reference No.: gc028_EA
Name of Compound: 9,9'-(5-(6-methylpyridazin-3-yl)-1,3-phenylene)bis(9H-carbazole)
Molecular Formula: C35H24N4
Stability: Air stable
Hazards: none
Other Remarks:

Element	Expected %	Found (1)	Found (2)		
Carbon	C, 83.98	83.86	83.88	83.87 av	0.11
Hydrogen	H, 4.83	4.90	4.92	4.91 av	0.09
Nitrogen	N, 11.19	11.03	11.12	11.075 av	0.115

Authorising Signature:

Date Completed: 250817	Signature: [illegible]
Comments:	

Figure **S15**. Elemental analysis results of **dCzMePydz**

LONDON metropolitan university

Elemental Analysis Service

Please send completed form and samples to:

Stephen Boyer
School of Human Sciences
Science Centre
London Metropolitan University
29 Hornsey Road
London N7 7DD

Telephone: 020 7133 3605
Fax: 020 7133 2577
Email: s.boyer@londonmet.ac.uk

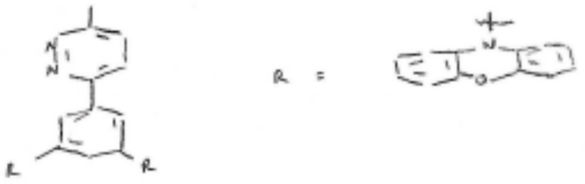

Sample submitted by: Graeme Copley	
Address: **EZC group, School of Chemistry, University of St Andrews, North Haugh,St Andrews, Fife, KY16 9ST**	
Telephone: 07931 680962	Email: gjc4@st-andrews.ac.uk
Date Submitted: 29/11/2017	

Please submit ca. 5 mg of sample.

Sample Reference No.:	gc025-EA1
Name of Compound:	
Molecular Formula:	$C_{35}H_{24}N_4O_2$
Stability: Air stable	
Hazards: none	
Other Remarks:	

Element	Expected %	Found (1)	Found (2)		
Carbon	78.93	78.96	79.04	79 =	0.07
Hydrogen	4.54	4.63	4.67	4.65 =	0.11
Nitrogen	10.52	10.43	10.44	10.435 =	0.085

Authorising Signature:

Date Completed: 05/12/17	Signature: [illegible]
Comments:	

Figure **S16**. Elemental analysis results of **dPXZMePydz**

LONDON metropolitan university

Elemental Analysis Service

Please send completed form and samples to:

Stephen Boyer
School of Human Sciences
Science Centre
London Metropolitan University
29 Hornsey Road
London N7 7DD

Telephone: 020 7133 3605
Fax: 020 7133 2577
Email: s.boyer@londonmet.ac.uk

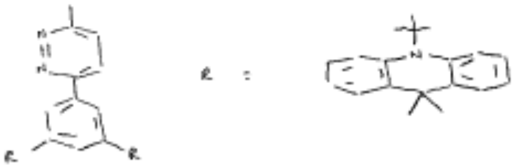

Sample submitted by: Graeme Copley	
Address: **EZC group, School of Chemistry, University of St Andrews, North Haugh,St Andrews, Fife, KY16 9ST**	
Telephone: 07931680962	Email: gjc4@st-andrews.ac.uk
Date Submitted: 12/12/2017	

Please submit ca. 5 mg of sample.

Sample Reference No.: gc033_EA
Name of Compound: 10,10'-(5-(6-methylpyridazin-3-yl)-1,3-phenylene)bis(9,9-dimethyl-9,10-dihydroacridine)
Molecular Formula: C41H36N4
Stability: Air stable
Hazards: none
Other Remarks:

Element	Expected %	Found (1)	Found (2)		
Carbon	C, 84.21	84.17	84.11	84.14 av	0.07
Hydrogen	H, 6.21	6.30	6.32	6.31 av	0.10
Nitrogen	N, 9.58	9.52	9.52	9.52 av	0.06

Authorising Signature:

Date Completed: 151217	Signature: [signature]
Comments:	

Figure **S17**. Elemental analysis results of **dPXZMePydz**

X-ray crystallography

X-ray diffraction data for **dPXZMePydz** were collected at 93 K, and for **dDMACMePydz** at 173 K, using a Rigaku FR-X Ultrahigh Brilliance Microfocus RA generator/confocal optics with XtaLAB P200 diffractometer [Mo Kα radiation (λ = 0.71075 Å)]. Intensity data were collected using ω steps accumulating area detector images spanning at least a hemisphere of reciprocal space. Data for both compounds were collected and processed (including correction for Lorentz, polarization and absorption) using CrystalClear.[3] Structures were solved by direct methods (SIR2011[4]) and refined by full-matrix least-squares against F^2 (SHELXL-2018/3[5]). Non-hydrogen atoms were refined anisotropically, and hydrogen atoms were refined using a riding model. All calculations were performed using the CrystalStructure[6] interface. Selected crystallographic data are presented in Table **S1**. Deposition numbers 2002948-2002949 contains the supplementary crystallographic data for this paper. These data are provided free of charge by the joint Cambridge Crystallographic Data Centre and Fachinformationszentrum Karlsruhe Access Structures service www.ccdc.cam.ac.uk/structures.

Table **S1**. Selected crystallographic data.

	**dPXZMePydz**	**dDMACMePydz**
empirical formula	$C_{35}H_{24}N_4O_2$	$C_{41}H_{36}N_4$
fw	532.60	584.76
crystal description	yellow prism	colourless prism
crystal size [mm^3]	0.29×0.21×0.04	0.19×0.06×0.03
space group	$P\bar{1}$	*Pbca*
a [Å]	10.5422(15)	31.601(5)
b [Å]	14.7792(16)	16.404(2)
c [Å]	18.041(3)	12.0596(14)
α [°]	71.017(7)	
β [°]	87.8915(10)	

γ [°]	75.667(9)	
vol [Å]^3	2572.3(6)	6251.5(14)
Z	4	8
ρ (calc) [g/cm^3]	1.375	1.243
μ [mm^{-1}]	0.087	0.073
F(000)	1112	2480
reflns collected	34323	72229
independent reflns (R_{int})	9253 (0.0641)	5721 (0.0629)
data/restraints/params	9253/0/741	5721/0/411
GOF on F^2	0.931	1.023
R_1 [$I > 2\sigma(I)$]	0.0410	0.0404
wR_2 (all data)	0.1191	0.1004
largest diff. peak/hole [e/Å^3]	0.31, -0.22	0.19, -0.18

Theoretical calculations

All ground state optimizations have been carried out at the Density Functional Theory (DFT) level with Gaussian09 software[7] employing the PBE0 functional[8] with the standard Pople 6-31G (d,p) basis set in vacuum, followed by the frequency calculations to ensure that the global energy minima was reached. For **dPXZMePydz** and **dDMACMePydz**, the crystal structures were used for the input geometries while for **dCzMePydz**, the geometry was optimized from a best-guest initial structure drawn in Gaussview.. Excited state calculations were performed using Time-Dependent DFT (TD-DFT) within the Tamm-Dancoff approximation (TDA)[9,10] using the same functional and basis set as for ground state geometry optimization. GaussSum software[11] was employed in visualizing the electronic transitions, while molecular orbitals were generated with GaussView software.[7]

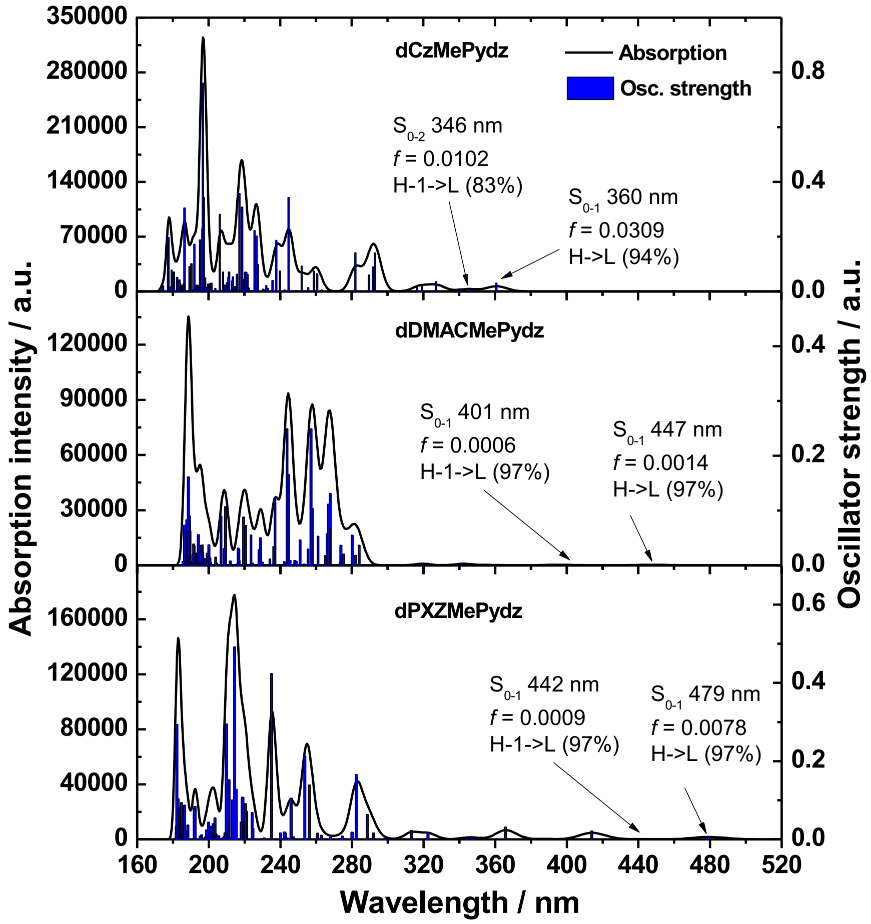

Figure **S18**. TDA simulation [PBE0/6-31G(d,p) in the gas phase] of UV-Vis spectra of **dCzMePydz**, **dDMACMePydz** and **dPXZMePydz**.

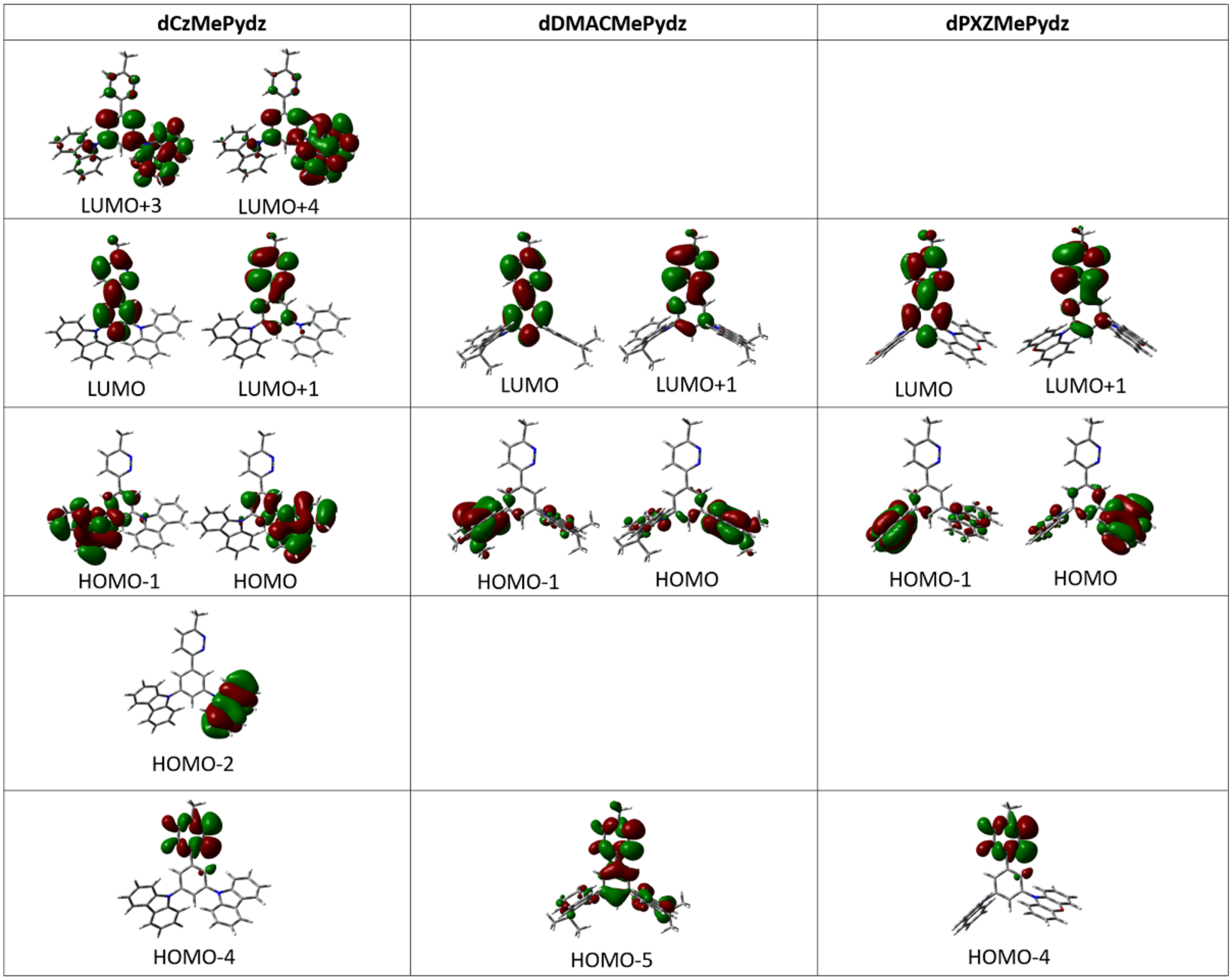

Figure **S19**. The dominant molecular orbitals of S_0-$S_{1,2}$ and S_0-$T_{1,2,3,4}$ excitations.

Table **S2**. Excitation energy, singlet-triplet gap ΔE_{ST}, oscillator strength, and composition of molecular orbital transitions.

**Compound**	**Excited state**	**Energy / eV**	**O.S. / a.u.**	**Main MO component of the transition from S_0**
**dPXZMePydz**	T_1	2.511	--	HOMO → LUMO (94%)
	S_1	2.587	$8.0 \cdot 10^{-3}$	HOMO → LUMO (97%)
	ΔE_{ST}	0.076		
	T_2	2.758		HOMO-1 → LUMO (92%)
	T_3	2.790		HOMO-4 → LUMO (35%),

				HOMO-4 → LUMO+1 (57%)
	S_2	2.799	$1.0 \cdot 10^{-3}$	HOMO-1 → LUMO (97%)
	ΔE_{S2T3}	0.009		
**dDMACMePydz**	T_1	2.739		HOMO → LUMO (96%)
	S_1	2.768	$1.4 \cdot 10^{-3}$	HOMO → LUMO (97%)
	ΔE_{ST}	0.029		
	T_2	2.803		HOMO-5 → LUMO (33%),<br>HOMO-5 → LUMO+1 (51%)
	ΔE_{S1T2}	-0.035		
	T_3	3.058		HOMO-1 → LUMO (94%)
	S_2	3.091	$6.0 \cdot 10^{-4}$	HOMO-1 → LUMO (97%)
	ΔE_{S2T3}	0.033		
**dCzMePydz**	T_1	2.801		HOMO-4 → LUMO+1 (53%)<br>HOMO-4 → LUMO (40%)
	S_1	3.436	$3.1 \cdot 10^{-2}$	HOMO → LUMO (94%)
	ΔE_{ST}	0.635		
	T_2	3.192		HOMO → LUMO (67%)
	T_3	3.264		HOMO-1 → LUMO (57%),<br>HOMO → LUMO (13%)
	T_4	3.451		HOMO-2 → LUMO+3 (37%),<br>HOMO-2 → LUMO+4 (42%)
	S_2	3.577	$1.02 \cdot 10^{-2}$	HOMO-1 → LUMO (83%)
	ΔE_{S1T4}	-0.015		

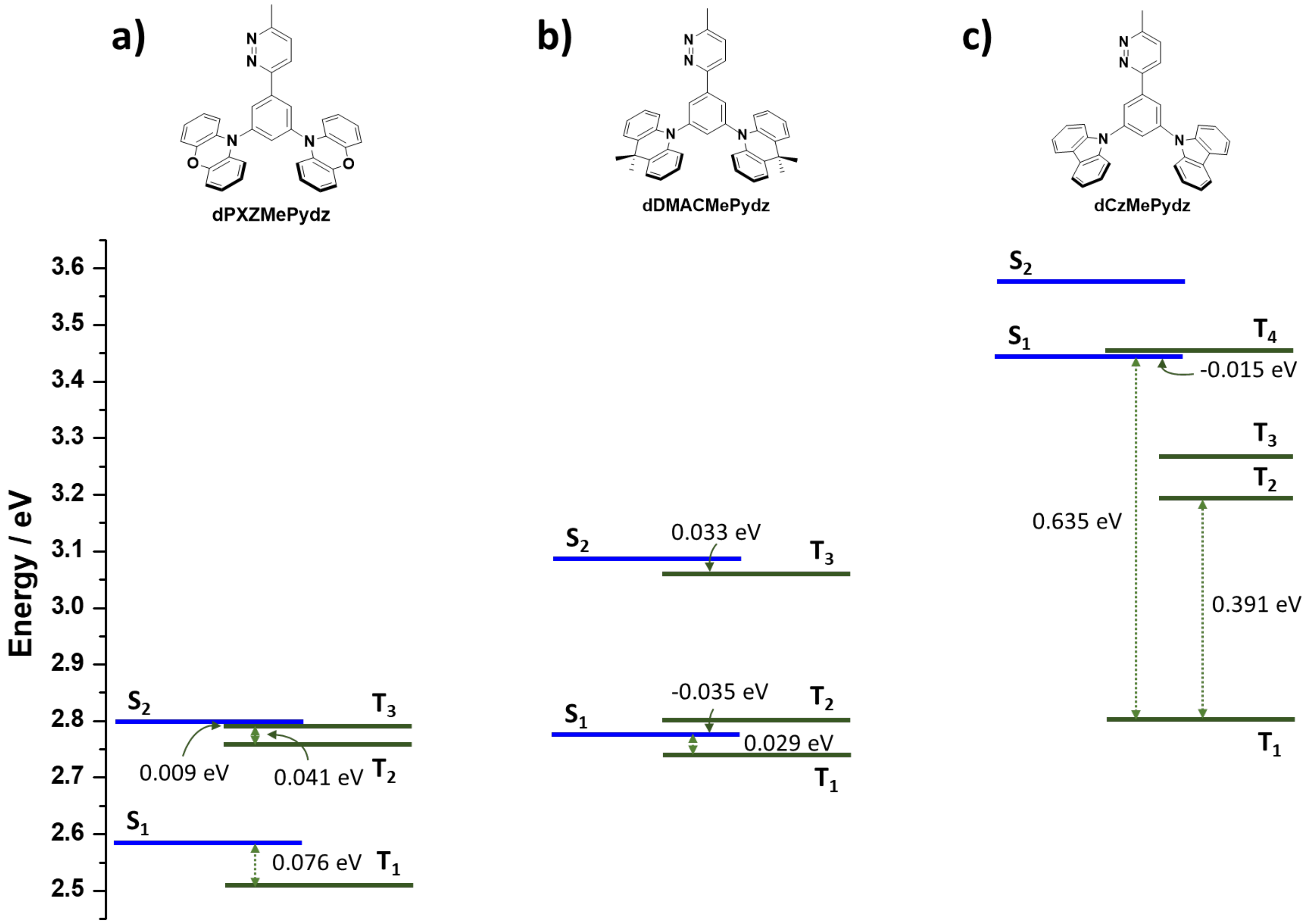

Figure **S20**. DFT predicted excited state energies and relevant energy gaps.

Photophysical measurements

Optically dilute solutions of concentrations on the order of 10^{-5} or 10^{-6} M were prepared in HPLC grade toluene, DCM and MeCN for absorption and emission analysis. Absorption spectra were recorded at room temperature on a Shimadzu UV-1800 double beam spectrophotometer with a 1 cm quartz cuvette. Molar absorptivity values were determined from at least four solutions followed by linear regression analysis.

For solution-state emission studies, aerated solutions were bubbled with compressed air for 5 minutes and spectra were taken using the same cuvette used for absorption analysis. Degassed solutions were prepared via five freeze-pump-thaw cycles and spectra were taken using a home-made Schlenk quartz cuvette. Steady-state emission, excitation spectra and time-resolved emission spectra were recorded at 298 K using an Edinburgh Instruments

F980. Samples were excited at 315, 330 or 360 nm for steady state measurements while at 378 nm for time-resolved measurements. Photoluminescence quantum yields for solutions were determined using the optically dilute method in which four sample solutions with absorbances of ca. 0.10, 0.080, 0.060 and 0.040 at 360 nm were used. The Beer-Lambert law was assumed to remain linear at the concentrations of the solutions. For each sample, linearity between absorption and emission intensity was verified through linear regression analysis with the Pearson regression factor (R^2) for the linear fit of the data set surpassing 0.9. Individual relative quantum yield values were calculated for each solution and the values reported represent the slope obtained from the linear fit of these results. The equation $\Phi_s = \Phi_r(A_r/A_s)(I_s/I_r)(n_s/n_r)^2$ was used to calculate the relative quantum yield of the sample, where (Φ_r) is the absolute quantum yield of the external reference quinine sulfate (Φ_r = 54.6% in 1 *N* H_2SO_4), A stands for the absorbance at the excitation wavelength, I is the integrated area under the corrected emission curve and n is the refractive index of the solvent. The subscripts "s" and "r" representing sample and reference, respectively. Thin film PLQY measurements were performed using an integrating sphere in a Hamamatsu C9920-02 system.[12] A xenon lamp coupled to a monochromator enabled excitation selectivity, chosen here to be 378 nm. The output was then fed into the integrating sphere via a fibre, exciting the sample. PL spectra were collected with a multimode fibre and detected with a back-thinned CCD. Doped thin films were prepared either by mixing sample (10 wt%) and PMMA in DCM solution, followed by spin-casting on a quartz substrate or by co-evaporation of sample (15 wt%) with DPEPO host in high vacuum. The Φ_{PL} of the films were then measured in air and by purging the integrating sphere with flowing N_2 gas. Time-resolved PL measurements of the thin films were carried out using the time-correlated single-photon counting technique. The samples were excited at 378 nm by a pulsed laser diode (Picoquant, model PLS 370) and were kept in a vacuum of $<8 \times 10^{-4}$ mbar.

The singlet-triplet splitting energy ΔE_{ST} was estimated by recording the prompt fluorescence spectra and phosphorescence emission at 77 K. The PMMA films were excited either by a Q-switched Nd:YAG laser emitting at 355 nm (Laser-export). Emission from the samples was focused onto a spectrograph (Chromex imaging, 250is spectrograph) and detected on a sensitive gated iCCD camera (Stanford Computer Optics, 4Picos) having sub-

nanosecond resolution. Phosphorescence spectra were measured 1 ms after the excitation of the Nd:YAG laser with iCCD exposure time of 9 ms. Prompt fluorescence spectra were measured 1 ns after the excitation of the femtosecond laser with iCCD exposure time of 10 ns.

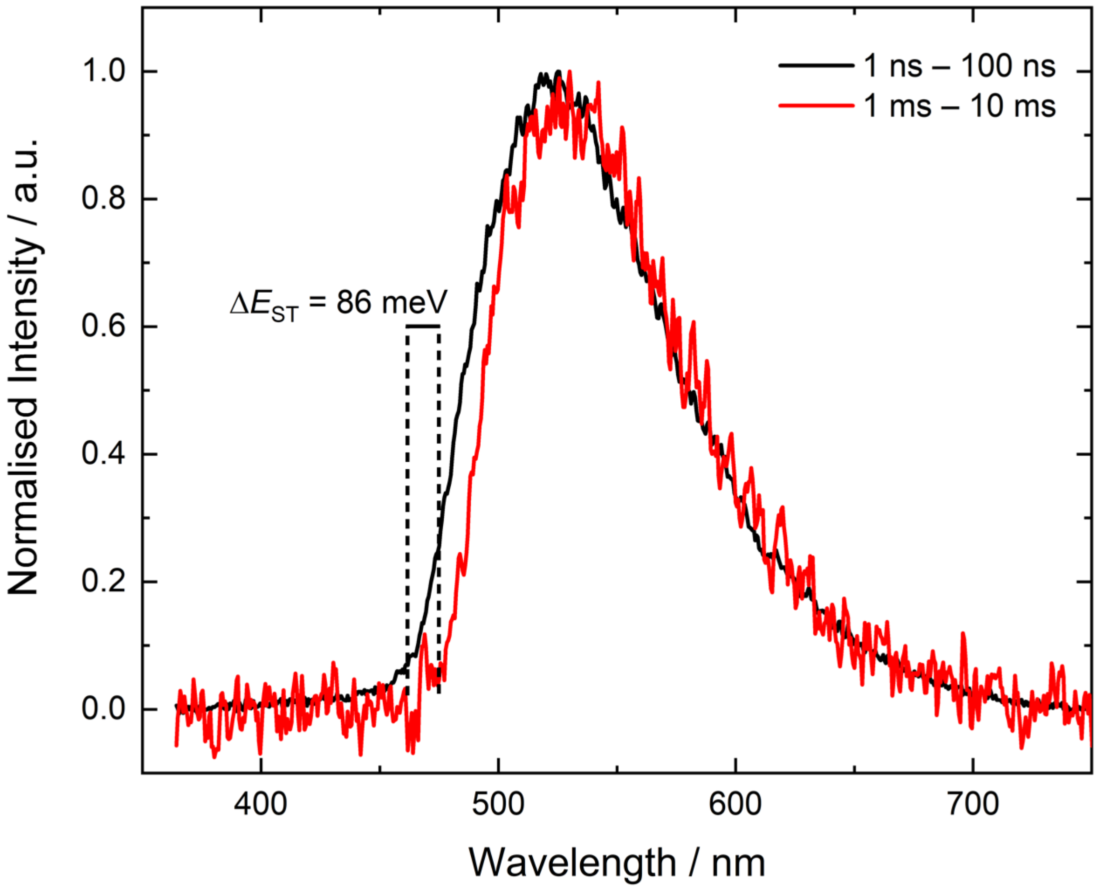

Figure **S21**. Prompt fluorescence and phosphorescence spectra of PMMA film doped with 10 wt% **dPXZMePydz** integrated over 1 ns – 100 ns and 1 ms – 10 ms after excitation (λ_{exc} = 355 nm).

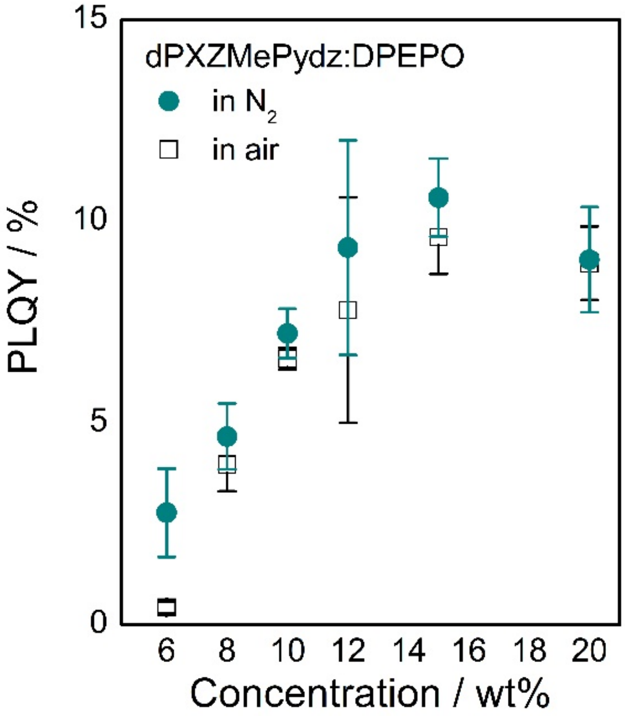

Figure **S22**. Thin film (40 nm thickness) Φ_{PL} for various concentrations of **dPXZMePydz** in DPEPO host.

Table S3. TADF parameters of **dPXZMePydz** doped into DPEPO host (15 wt% concentration).

Compound	Φ_{DF} / Φ_{PF} [a]	Φ_{ISC} / % [b]	k_{ISC} / s^{-1} [c]	k_{rISC} / s^{-1} [d]
**dPXZMePydz**	4.6	82	$2.0 \cdot 10^7$	$3.9 \cdot 10^6$

[a]Delayed fluorescence to prompt fluorescence yield ratio. [b]Triplet yield. [c]Intersystem crossing rate. [d]Reverse intersystem crossing rate.

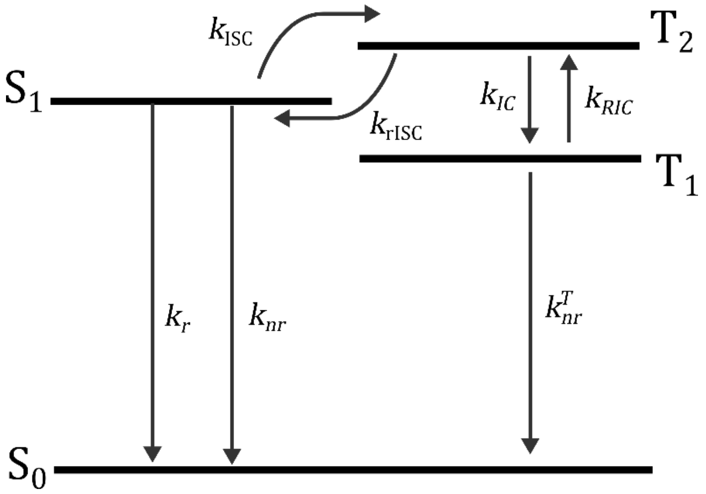

Figure **S23**. Energy level diagram and corresponding rates used for **dDMACMePydz**:DPEPO thin film PL analysis.

Table S4. Four energy level analysis parameters for **dDMACMePydz**:DPEPO.

k_r / s^{-1} [a]	Φ_{FL} / % [b]	Φ_{ISC} / % [c]	Φ_{rISC} / % [d]
$1.21 \cdot 10^6$	1.3	98.7	1.1
k_{ISC} / s^{-1}	k_{rISC} / s^{-1}	k_{IC} / s^{-1}	k_{RIC} / s^{-1}
$9.21 \cdot 10^7$	$1.37 \cdot 10^7$	$1.52 \cdot 10^7$	$1.01 \cdot 10^5$

[a]Radiative decay rate. [b]Fluorescence yield. [c]Triplet yield. [d]Triplet conversion yield.

Electrochemistry measurements

Cyclic Voltammetry (CV) analysis was performed on an Electrochemical Analyzer potentiostat model 620E from CH Instruments at a sweep rate of 100 mV/s. Differential pulse voltammetry (DPV) was conducted with an increment potential of 0.004 V and a pulse amplitude, width, and period of 50 mV, 0.05, and 0.5 s, respectively. Samples were prepared as either dichloromethane (DCM, used for oxidation) or dimethylformamide (DMF, used for reduction) solutions, which were degassed by sparging with solvent-saturated argon gas for 15 minutes prior to measurements. All measurements were performed using 0.1 M DCM or DMF solutions of tetra-*n*-butylammonium hexafluorophosphate ([$n$$Bu_4N$]$PF_6$). An Ag/Ag^+ electrode was used as the reference electrode, while a platinum electrode and a platinum wire were used as the working electrode and counter electrode,

respectively. The redox potentials are reported relative to a saturated calomel electrode (SCE) with a ferrocenium/ferrocene (Fc/Fc^+) redox couple as the internal standard (0.46 V vs SCE for DCM and 0.45 V vs SCE for DMF).[13]

OLED fabrication and characterization

The OLED devices were fabricated in bottom-emitting architecture via vacuum sublimation in high vacuum at a base pressure of $2\text{-}5\cdot10^{-7}$ mbar. The organic layer sequence and the metal cathode were deposited onto pre-cleaned glass substrates coated with indium tin oxide (ITO) which has a sheet resistance of around 30 Ω/sq. A pre-patterned ITO glass substrate was treated by ultrasonic cleaning in acetone and *iso*propanol consecutively and then treated by oxygen plasma before the transfer to the vacuum chamber. Organic layers were deposited at a rate of 0.3-0.6 Å/s, which was controlled *in situ* using the quartz crystal monitors. Four different device architectures were tested, with the general functional layer sequence: ITO (90 nm)/TAPC (35 nm)/mCP (10 nm)/ **Emitter**:Host (15wt%, 30 nm)/TPBi (x nm)/LiF (1 nm)/Al (100 nm). Here x = 45 nm for **dPXZMePydz** emitter and x = 55 nm for **dDMACMePydz**. DPEPO and 3,3′-di(9H-carbazol-9-yl)-1,1′-biphenyl (mCBP) were employed as host materials. Results of the best performing devices are summarized in Table **S5**. Doping of the emission layers was achieved through co-evaporation of the emitter and host materials. The electron injection layer LiF was deposited at a rate of 0.05 Å/s while the Al cathode was deposited at a rate of 2 Å/s through the shadow mask defining the top electrode. The spatial overlap of the anode and cathode electrodes determined the active area of the OLED, which was estimated to be 2 and 16 mm^2.

All the devices were encapsulated within UV epoxy resin under an inert atmosphere. The luminance-current-voltage characteristics were measured in the ambient environment using a Keithley 2400 source meter and Keithley 2000 multimeter connected to a calibrated Si photodiode. The external quantum efficiency was calculated assuming Lambertian emission distribution. The electroluminescence spectra were recorded by an Andor DV420-BV CCD spectrometer.

Table S6. Best performance of OLEDs with different emitter/host combinations. Emitter concentration is kept at 15 wt%.

Host	Emitter [a]	V_{on} / V [b]	λ_{EL} / nm	CIE	EQE / % [c]	PE / lm W^{-1} [d]
DPEPO	**dPXZMePydz**	3.5	523	(0.30, 0.56)	6.4 / 5.7 / 4.6	11.4
mCBP	**dPXZMePydz**	3.8	520	(0.29, 0.50)	4.0 / 3.9 / 3.8	7.3
DPEPO	**dDMACMePydz**	4.1	489	(0.18, 0.33)	0.5 / 0.5 / 0.4	0.4
mCBP	**dDMACMePydz**	5.1	469	(0.17, 0.22)	0.4 / 0.4 / 0.3	0.2

[a]At 15 wt% concentration. [b]Defined as voltage at 1 cd m^{-2} brightness. [c]Maximal value / at 100 cd m^{-2} / at 1000 cd m^{-2}. [d]At 100 cd m^{-2}.

Cartesian coordinates

Optimized Atomic coordinates obtained from DFT calculations of the ground state geometries of **dPXZMePydz, dDMACMePydz** and **dCzMePydz:**

dPXZMePydz

Row	Tag	Symbol	X	Y	Z
1	1	O	-3.65185	-3.67868	0.010648
2	2	O	5.266217	-0.42503	0.57257
3	3	N	-1.90182	-1.47507	-0.00293
4	4	N	2.593794	0.363324	0.252004
5	5	N	-2.54597	3.507156	-0.31056
6	6	N	-3.15245	4.661375	-0.50376
7	7	C	3.137872	0.222373	1.537296
8	8	C	4.650805	-0.52899	-0.64921
9	9	C	4.476675	-0.17365	1.66731
10	10	C	-2.86736	-1.608	1.006632
11	11	C	2.404707	0.478511	2.694134
12	12	H	1.367283	0.782129	2.605891
13	13	C	-1.78534	-2.47927	-0.97676
14	14	C	5.060291	-0.30763	2.914171
15	15	H	6.098066	-0.62067	2.96091
16	16	C	-3.72619	-2.71667	0.98498
17	17	C	2.994633	0.350605	3.950578
18	18	H	2.403849	0.559211	4.836663
19	19	C	5.404137	-1.02201	-1.69956
20	20	H	6.426849	-1.32206	-1.49664

21	21	C	3.53809	-0.71883	-3.1821
22	22	H	3.092173	-0.7821	-4.16958
23	23	C	-1.53583	0.915932	-0.27137
24	24	H	-2.59868	1.064011	-0.42743
25	25	C	-2.46986	5.71792	-0.95558
26	26	C	0.69214	1.826499	-0.13182
27	27	H	1.393682	2.653664	-0.15126
28	28	C	4.850523	-1.11815	-2.97591
29	29	H	5.448515	-1.50215	-3.79557
30	30	C	-1.03263	-0.35881	-0.0235
31	31	C	2.776171	-0.23017	-2.12162
32	32	H	1.753984	0.091507	-2.2886
33	33	C	-1.24258	3.363951	-0.57004
34	34	C	-2.668	-3.56952	-0.93711
35	35	C	-4.824	-1.96308	2.991684
36	36	H	-5.5866	-2.10316	3.750447
37	37	C	-2.99598	-0.69719	2.05466
38	38	H	-2.33218	0.158351	2.101268
39	39	C	4.32095	-0.04051	4.065801
40	40	H	4.785715	-0.14293	5.040709
41	41	C	0.334416	-0.54245	0.180644
42	42	H	0.731921	-1.53305	0.37609
43	43	C	-2.59241	-4.58486	-1.87425
44	44	H	-3.29886	-5.40415	-1.79145
45	45	C	-3.97075	-0.87033	3.035362
46	46	H	-4.05184	-0.13942	3.833332
47	47	C	-0.84658	-2.4352	-2.00779
48	48	H	-0.16443	-1.59614	-2.07377
49	49	C	-1.10433	5.633412	-1.25625
50	50	H	-0.56989	6.49719	-1.64087
51	51	C	-0.47346	4.426018	-1.0666
52	52	H	0.575035	4.295008	-1.31263
53	53	C	-3.24725	6.983901	-1.13015
54	54	H	-2.84732	7.781992	-0.49585
55	55	H	-4.2893	6.807624	-0.86067
56	56	H	-3.20199	7.335173	-2.16623
57	57	C	-1.63833	-4.53714	-2.88878
58	58	H	-1.58422	-5.33815	-3.61836
59	59	C	-0.77069	-3.45705	-2.95228
60	60	H	-0.02321	-3.39459	-3.73658
61	61	C	3.319411	-0.13234	-0.84111
62	62	C	-4.69194	-2.89082	1.960305
63	63	H	-5.33071	-3.76513	1.891993
64	64	C	1.192109	0.551148	0.10352
65	65	C	-0.68072	2.020699	-0.31545

dDMACMePydz

Row	Tag	Symbol	X	Y	Z
1	1	N	2.454355	-0.61271	0.083921
2	2	N	-2.44158	-0.5658	0.080883
3	3	N	-1.28768	5.590642	-0.75623
4	4	N	-1.16179	4.269849	-0.61533
5	5	C	3.247638	-0.58972	1.254679
6	6	C	0.014102	3.723642	-0.22278
7	7	C	1.234336	1.52071	-0.05509
8	8	H	2.20281	2.021122	-0.13079
9	9	C	3.05348	-0.94363	-1.1552
10	10	C	1.223033	0.121979	0.060938
11	11	C	0.010004	-0.57343	0.120298
12	12	H	0.003149	-1.66219	0.207321
13	13	C	-3.10158	-0.72561	1.312387
14	14	C	-1.1915	1.536421	-0.06622
15	15	H	-2.13081	2.089496	-0.12863
16	16	C	4.229182	-1.73634	-1.15602
17	17	C	1.139561	4.52525	0.06617
18	18	H	2.074133	4.085304	0.424077
19	19	C	2.881185	0.188612	2.371724
20	20	H	1.949558	0.757196	2.355981
21	21	C	-2.95211	-1.10141	-1.11593
22	22	C	4.670008	-2.37654	0.161616
23	23	C	3.711318	0.241631	3.49574
24	24	H	3.411947	0.851816	4.352828
25	25	C	5.258416	-1.26907	2.417882
26	26	H	6.185256	-1.84639	2.45301
27	27	C	1.012288	5.896393	-0.09505
28	28	H	1.847143	6.572589	0.113088
29	29	C	4.916977	-0.46807	3.516176
30	30	H	5.578344	-0.41884	4.385171
31	31	C	-0.23216	6.404358	-0.51917
32	32	C	-4.18428	-1.79887	-1.1212
33	33	C	4.334963	-1.50878	-3.59363
34	34	H	4.845107	-1.72781	-4.53531
35	35	C	2.507805	-0.48352	-2.37113
36	36	H	1.59579	0.116316	-2.36567
37	37	C	3.144103	-0.77407	-3.5817
38	38	H	2.70817	-0.40856	-4.51589
39	39	C	-2.52034	-0.20761	2.493262
40	40	H	-1.56127	0.310014	2.437302

41	41	C	-4.33998	-1.40809	1.378819
42	42	C	4.860824	-1.9822	-2.38375
43	43	H	5.777851	-2.57579	-2.40187
44	44	C	-4.95452	-1.53219	2.636416
45	45	H	-5.91501	-2.0531	2.698927
46	46	C	-5.02733	-1.99924	0.142966
47	47	C	-0.47371	7.873495	-0.72463
48	48	H	-1.5199	8.032161	-1.0202
49	49	H	0.184421	8.278236	-1.51209
50	50	H	-0.26997	8.444648	0.196593
51	51	C	-2.23358	-0.93687	-2.32304
52	52	H	-1.28752	-0.39293	-2.31793
53	53	C	-3.15911	-0.35129	3.725589
54	54	H	-2.6882	0.059565	4.623276
55	55	C	3.741493	-3.59965	0.422813
56	56	H	3.85209	-4.34029	-0.38635
57	57	H	2.685508	-3.29105	0.465138
58	58	H	4.000937	-4.07432	1.38345
59	59	C	-2.72157	-1.45886	-3.52152
60	60	H	-2.14443	-1.31783	-4.43983
61	61	C	6.122615	-2.87299	0.115378
62	62	H	6.830955	-2.04865	-0.06484
63	63	H	6.250145	-3.62495	-0.67808
64	64	H	6.39365	-3.36857	1.060023
65	65	C	-4.38859	-1.01611	3.806794
66	66	H	-4.90064	-1.13352	4.765258
67	67	C	-3.93786	-2.15175	-3.54317
68	68	H	-4.33275	-2.5634	-4.47548
69	69	C	-5.25097	-3.51796	0.367472
70	70	H	-4.28803	-4.03391	0.503985
71	71	H	-5.76723	-3.97373	-0.49225
72	72	H	-5.86806	-3.6979	1.261907
73	73	C	-4.64604	-2.30713	-2.3471
74	74	H	-5.60001	-2.84277	-2.36405
75	75	C	-6.39772	-1.29703	-0.05205
76	76	H	-6.9295	-1.70824	-0.92507
77	77	H	-6.25856	-0.21664	-0.21165
78	78	H	-7.03969	-1.43448	0.83294
79	79	C	0.028282	2.244656	-0.10572
80	80	C	-1.19651	0.142316	0.045984
81	81	C	4.431418	-1.37086	1.289818

dCzMePydz

Row	Tag	Symbol	X	Y	Z
1	1	C	-0.27531	3.467319	-0.05656
2	2	C	0.776271	4.327284	-0.40366
3	3	C	0.533589	5.680114	-0.35428
4	4	C	-0.74677	6.106122	0.022436
5	5	H	1.738812	3.937557	-0.71872
6	6	H	1.301875	6.403465	-0.61158
7	7	C	-1.11159	7.554492	0.105601
8	8	H	-2.16224	7.646735	0.383518
9	9	H	-0.50105	8.071432	0.853501
10	10	H	-0.95115	8.058146	-0.85325
11	11	C	-0.13285	1.996587	-0.05446
12	12	C	1.118106	1.394334	0.086921
13	13	C	-1.2725	1.199717	-0.1813
14	14	C	1.227328	0.004496	0.086973
15	15	H	2.013549	1.986412	0.241185
16	16	C	-1.1621	-0.18829	-0.15234
17	17	H	-2.23792	1.675382	-0.30643
18	18	C	0.09052	-0.79228	-0.02469
19	19	H	0.179748	-1.87255	-0.00703
20	20	N	-1.7146	5.233734	0.319818
21	21	N	-1.47898	3.937	0.286084
22	22	C	-3.48379	-0.82625	0.478147
23	23	C	-2.46514	-2.08597	-1.10576
24	24	C	-3.78838	0.110104	1.464428
25	25	C	-4.3959	-1.84114	0.110676
26	26	C	-1.58982	-2.60007	-2.06054
27	27	C	-3.7479	-2.64263	-0.90065
28	28	C	-5.03791	0.024268	2.065473
29	29	H	-3.08361	0.88202	1.753413
30	30	C	-5.64417	-1.90431	0.730889
31	31	C	-2.01006	-3.70483	-2.79067
32	32	H	-0.61897	-2.15106	-2.23923
33	33	C	-4.1452	-3.752	-1.64767
34	34	C	-5.95998	-0.96706	1.70407
35	35	H	-5.30231	0.745845	2.832428
36	36	H	-6.35436	-2.67896	0.456492
37	37	C	-3.2703	-4.28167	-2.58575
38	38	H	-1.34502	-4.12556	-3.53879
39	39	H	-5.12839	-4.18902	-1.49898
40	40	H	-6.92837	-1.00125	2.193214
41	41	H	-3.56531	-5.146	-3.17234
42	42	C	2.840982	-1.56168	1.166435
43	43	C	3.61182	-0.30251	-0.54832

44	44	C	2.068151	-2.13441	2.174526
45	45	C	4.203321	-1.89635	0.992521
46	46	C	3.735919	0.567744	-1.62964
47	47	C	4.695263	-1.0936	-0.10245
48	48	C	2.67963	-3.07173	2.997315
49	49	H	1.029986	-1.85557	2.319336
50	50	C	4.792429	-2.84138	1.833542
51	51	C	4.977547	0.653005	-2.24705
52	52	H	2.890833	1.147552	-1.98571
53	53	C	5.931699	-0.98786	-0.74056
54	54	C	4.02473	-3.42795	2.829527
55	55	H	2.099368	-3.5352	3.789397
56	56	H	5.837981	-3.1088	1.710927
57	57	C	6.067545	-0.11015	-1.80715
58	58	H	5.101812	1.322756	-3.09276
59	59	H	6.773111	-1.58915	-0.40861
60	60	H	4.468898	-4.16635	3.489493
61	61	H	7.024072	-0.01735	-2.3117
62	62	N	2.493635	-0.59415	0.226864
63	63	N	-2.31327	-0.98648	-0.26329

References

1 J. Kaleta, E. Kaletová, I. Císařová, S. J. Teat and J. Michl, *J. Org. Chem.*, 2015, **80**, 10134–10150.

2 T. H. Kwon, M. K. Kim, J. Kwon, D. Y. Shin, S. J. Park, C. L. Lee, J. J. Kim and J. I. Hong, *Chem. Mater.*, 2007, **19**, 3673–3680.

3 *CrystalClear-SM Expert v.*2.1. Rigaku Americas, Rigaku Americas, The Woodlands, Texas, USA and Rigaku Corporation, Tokyo, Japan, 2015.

4 M. C. Burla, R. Caliandro, M. Camalli, B. Carrozzini, G. L. Cascarano, C. Giacovazzo, M. Mallamo, A. Mazzone, G. Polidori and R. Spagna, *J. Appl. Crystallogr.*, 2012, **45**, 357–361.

5 G. M. Sheldrick, *Acta Crystallogr. Sect. C Struct. Chem.*, 2015, **71**, 3–8.

6 *CrystalStructure v.*4.3.0. Rigaku Americas, The Woodlands, Texas, USA, and Rigaku Corporation,

Tokyo, Japan, 2018.

7 M. J. Frisch, G. W. Trucks, H. B. Schlegel, G. E. Scuseria, M. A. Robb, J. R. Cheeseman, G. Scalmani, V. Barone, B. Mennucci, G. A. Petersson, H. Nakatsuji, M. Caricato, X. Li, H. P. Hratchian, A. F. Izmaylov, J. Bloino, G. Zheng, J. L. Sonnenberg, M. Hada and M. D. J. Ehara, *Gaussian 09, Revis. A.02*, 2009.

8 J. A. Pople, J. S. Binkley and R. Seeger, *Int. J. Quantum Chem.*, 1976, **10**, 1–19.

9 S. Grimme, *Chem. Phys. Lett.*, 1996, **259**, 128–137.

10 J. Tomasi, B. Mennucci and R. Cammi, *Chem. Rev.*, 2005, **105**, 2999–3094.

11 N. M. O'Boyle, A. L. Tenderholt and K. M. Langner, *J. Comput. Chem.*, 2008, **29**, 839–845.

12 N. C. Greenham, I. D. W. Samuel, G. R. Hayes, R. T. Phillips, Y. A. R. R. Kessener, S. C. Moratti, A. B. Holmes and R. H. Friend, *Chem. Phys. Lett.*, 1995, **241**, 89–96.

13 N. G. Connelly and W. E. Geiger, *Chem. Rev.*, 1996, **96**, 877–910.
